# Signal peptide recognition in *Trypanosoma cruzi* GP82 adhesin relies on its localization at protein *N*-terminus

**DOI:** 10.1038/s41598-019-43743-0

**Published:** 2019-05-13

**Authors:** Esteban M. Cordero, Cristian Cortez, Nobuko Yoshida, José Franco da Silveira

**Affiliations:** 1 Departamento de Microbiologia, Imunologia, e Parasitologia, Escola Paulista de Medicina, Universidade Federal de São Paulo, São Paulo, Brazil; 2 Centro de Genómica y Bioinformática, Facultad de Ciencias, Universidad Mayor, Santiago, Chile

**Keywords:** Post-translational modifications, Parasitology, Protein transport

## Abstract

*Trypanosoma cruzi*, the causative agent of Chagas disease, has a dense coat of GPI-anchored virulence factors. *T*. *cruzi* GPI-anchored adhesin GP82 is encoded by a repertoire of transcripts containing several in-frame initiation codons located up-stream from that adjacent to the predicted signal peptide (SP). Transfection of *T*. *cruzi* epimastigotes with constructs encoding GP82 starting at the SP or from the farthest up-stream methionine confirmed protein expression on the parasite cell surface, comparable to the native GP82. Proteins were fully functional, inducing parasite adhesion to HeLa cells and lysosome mobilization, events required for parasite invasion. Transgenic and native GP82 proteins showed indistinguishable electrophoretic mobility, suggesting similar processing of the SP. Deletion of SP generated a ~72 kDa protein devoid of *N*-linked oligosaccharides allowing irrefutable identification of GP82 precursor. SP transposition to an internal region of GP82 rendered the signal unrecognizable by the signal peptidase and incapable to direct the nascent protein for ER-membrane association. Altogether our data strongly suggests that GP82 SP fails to function as transmembrane domain and its recognition by the signal peptidase shows strict dependence on the signal localization at protein N-terminus. This report presents the first experimental characterization of the full-length GP82 and its signal peptide.

## Introduction

Chagas disease is a neglected chronic illness with an estimate of 6–7 million individuals affected worldwide, mainly in Latin America where the disease is endemic and a major public health concern^1^. *Trypanosoma cruzi*, the etiological agent of Chagas disease, is a vector-borne flagellated protozoan parasite that circulates in the bloodstream of infected humans and mammalian reservoirs and invades several types of nucleated cells inside which it replicates by binary fission. Intracellular parasites differentiate into trypomastigotes, which are released to the circulation where they can spread the infection to other organs and tissues. The metacyclic trypomastigote (MT) surface glycoprotein GP82 is the main stage-specific virulence factor involved in the adhesion to and invasion of host cells^2,3^. MT is the infective stage found in the invertebrate vector and is transmitted to humans and other mammals during the insect blood meal. Additionally, MTs are involved in micro epidemics of acute Chagas disease acquired by consumption of contaminated food^4,5^. Orally ingested parasites can resist the acidic pH in the stomach, migrate through the gastric mucin layer and finally invade the underlying mucosal cells^6^. *In vitro*, GP82 binding to mammalian cells triggers responses such as Ca^++^ signalling and lysosome mobilization, both events required for successful parasite penetration^3^. *In vivo* studies demonstrated that GP82 is necessary for MT binding to the gastric mucin, a step required for targeting and invasion of gastric epithelial cells in the murine model of oral infection^7,8^. Additionally, immunization of mice with the recombinant protein containing the GP82 functional domains confers protection against acute *T*. *cruzi* infection^9,10^.

GP82 belongs to the *trans*-sialidase superfamily, the largest *T*. *cruzi* multigene family, encoding important virulence factors out of 1,430 members^11^. It is attached to the cell surface by a glycosylphosphatidylinositol (GPI) anchor^12^ a posttranslational modification conserved among eukaryotes^13^. Proteins that will acquire GPI anchor contain two signals in their primary structure, signal peptide (SP) and GPI-addition signal peptide (GPIsp), which are located at N- and C-terminus, respectively. SP drives the nascent proteins to the endoplasmic reticulum (ER), where it is co-translocationally removed by a signal peptidase. In *Trypanosoma brucei*, polytopic membrane proteins are targeted to the ER by the signal recognition particle (SRP)-receptor (SR) pathway while GPI-anchored proteins are translocated by an SRP-independent pathway^14–16^. The proteins continue to translocate into the ER lumen until the GPIsp stops the transference and the hydrophobic C-terminal domain is then cleaved and replaced by a preformed GPI-anchor. This exchange proceeds by a transamidation reaction catalysed by a multiprotein complex named transamidase^17^.

SPs are tripartite targeting signals arranged in the following order: a positively charged n-region, a central hydrophobic h-region and a terminal c-region composed of small polar amino acids. This latter region contains the cleavage site for the signal peptidase and is usually preceded by small neutral amino acids at the position −3 and −1^18^. Besides these common physicochemical features, there is no sequence conservation among SPs, hence the identification by sequence similarity is difficult^19^. Due to the variable nature of the hydrophobic region, SPs can be misclassified as transmembrane helices of a mature, membrane-spanning protein rather than an N-terminal membrane-spanning signal peptide which will be cleaved in ER^20^. Thus, experimental characterization is necessary to confirm if a predicted hydrophobic N-terminal region corresponds indeed to a cleavable SP or to an uncleaved transmembrane signal anchor. To the present date, only three proteins from *T*. *cruzi* TS superfamily had their mature N-terminus sequenced. The experimental data on the complement regulatory protein, CRP-10^21^, SAPA antigen^22^ and ASP-2^23^ demonstrated a processing of the primary translation product, compatible with the cleavage of a SP. Although this approach allowed the determination of the SP cleavage site, no data regarding the length of the primary translation product that originates those mature proteins have been reported^21–23^. In the case of CRP-10, for which the mature N-terminus is known^21^, it was suggested that the primary translation product initiates at the third starting codon, which is embedded in a Kozak context and encodes a canonical SP. The same criterion was applied to define the primary translation product of TSA-1, although its mature N-terminus was not known^24^, which would start at the SP (second ATG codon). Both TSA-1 and CRP-10 open reading frames contain additional in-frame starting codons located upstream from those adjacent to the SP encoding stretches of 37 and 38 amino acids, respectively^21,24^.

Although GP82 gene family is composed by a relatively small set of genes (19 complete sequences in *T*. *cruzi* CLB genome), its repertoire is quite variable^25–28^. Most GP82 genes have 2 start codons in the same reading frame, but only the 2^nd^ codon is inserted within the Kozak sequence context^26,28^, which could facilitate the mRNA translation^29^. After the 2^nd^ start codon there is a highly conserved hydrophobic sequence (M S R R V F/T S V L L L L F/L V), which could act as a SP addressing the nascent GP82 protein into the ER. Based on these findings, it was suggested that the translation initiates at the 2^nd^ start codon^26,28^. We have isolated a *T*. *cruzi* MT cDNA (GenBank EF154827) encoding a full-length GP82 protein that contains three in-frame methionines upstream from the SP sequence^26,28^. To study the GP82 trafficking and processing in *T*. *cruzi*, we generated tagged versions of GP82 protein on the backbone of this GP82 representative gene. Herein we describe the characterization of these proteins with special attention to the influence of those additional methionines in the processing of the SP.

## Results

### The presence of in-frame methionines up-stream from the SP is a common feature in *T*. *cruzi* virulence factors from TS multigene family

A considerable number of *T*. *cruzi* mRNAs encoding GPI-anchored virulence factors from TS multigene family contains at least one in-frame ATG start codon up-stream from the one adjacent to the SP. We searched the 5′ end of TS sequences for common sequences and/or structural features involved in the translation and processing of TS virulence factors. Table 1 shows the analysis of cDNAs and genomic DNA sequences from representative members of TS-superfamily containing additional up-stream ATG start codons (methionines), including stage-specific virulence factors involved in host-cell invasion, evasion of host immune response and induction of autoimmune response by molecular mimicry, such as, TS epi, SAPA, GP82, GP90, ASP-2, CRP, FL-160 and Tc85-11 proteins^3,21,30–37^. Many TS genes have 2–3 potential translation start sites in the same reading frame. Analysis of upstream sequences in the vicinity of the initiator codons showed that only the last ATG is in the context of the Kozak consensus sequence and is followed by the SP sequence. Some of these cDNA sequences contain a portion of the spliced-leader (SL) sequence at the 5′-end indicating that these molecules possess a full 5′-UTR. It is noteworthy that, among the GP82 sequences analysed, there is only one SL-containing cDNA member (EF154829) without additional ATG start codons up-stream from the SP (which means that the first ATG is adjacent to the SP), suggesting that this configuration although does exist, is underrepresented.

**Table 1:** Methionines up-stream from signal peptide in representative members of *T*. *cruzi* trans-sialidase (TS) superfamily.

GenBank Accession	Source	Strain/clone	Kozak consensus*	SplicedLeader (SL)**	Protein ID	In-frame Methionines***	Distance (aa)****
EF154827	cDNA	G	Yes	Yes	GP82	2	38
KJ189371	cDNA	G	Yes	Yes	GP82	2	38
KJ189375	cDNA	G	Yes	Yes	GP82	2	38
KJ189377	cDNA	G	Yes	Yes	GP82	2	38
KJ189381	cDNA	G	Yes	No	GP82	2	38
KY073275	cDNA	G	Yes	Yes	GP82	2	38
XM_806590	gDNA	CLB	Yes	No	GP82	2	38
XM_810099	gDNA	CLB	Yes	No	GP82	2	38
XM_810104	gDNA	CLB	Yes	No	GP82	2	38
XM_806594	gDNA	CLB	Yes	No	GP82	1	38
XM_815090	gDNA	CLB	Yes	No	GP82	1	38
XM_801751	gDNA	CLB	Yes	No	GP82	1	38
XM_811439	gDNA	CLB	Yes	No	GP82	1	38
XM_813769	gDNA	CLB	Yes	No	GP82	1	38
XM_800440	gDNA	CLB	Yes	No	GP82	1	38
XM_804566	gDNA	CLB	Yes	No	GP82	1	39
XM_807453	gDNA	CLB	Yes	No	GP82	1	38
XM_816676	gDNA	CLB	Yes	No	GP82	1	38
XM_802018	gDNA	CLB	Yes	No	GP82	1	38
XM_799595	gDNA	CLB	Yes	No	GP82	1	38
XM_805521	gDNA	CLB	Yes	No	GP82	1	30
XM_799474	gDNA	CLB	Yes	No	GP82	1	20
KR608067	cDNA	G	Yes	Yes	GP82	1	38
EF154828	cDNA^Ψ^	G	Yes	No	GP82	1	38
EF154829	cDNA	G	Yes	Yes	GP82	0	0
KJ189380	cDNA	G	Yes	No	GP82	0	0
XM_806823	gDNA	CLB	Yes	No	GP82	0	0
AF426132	cDNA	G	Yes	Yes	GP90	1	38
KY073276	cDNA	G	Yes	Yes	GP90	1	38
AF051696	cDNA	CL	Yes	Yes	P85.2 (GP85)	2	38
XM_808586	gDNA	CLB	Yes	No	Tc85-11 (GP85)	1	38
M58466	cDNA	Peru	Yes	Yes	TSA-1 (GP85)	1	37
AF051695	cDNA	CL	Yes	Yes	P85.1 (GP85)	1	38
CF889573	cDNA	CLB	Yes	Yes	EST (GP85)	1	38
EF579921	cDNA	Tulahuen	Yes	No	ASP-2	2	38
AY186573	cDNA	Y	Yes	Yes	ASP-2	1	38
U77951	cDNA	Brazil	Yes	Yes	ASP-2	1	38
AY186574	cDNA	Y	Yes	No	ASP-2	1	38
AY186574	cDNA	Y	Yes	No	ASP-2	1	38
EF583446	cDNA^Ψ^	Dm28c	Yes	No	ASP-2	1	38
EF579922	cDNA	G	Yes	No	ASP-2	1	38
GU445326	cDNA	Tulahuen	Yes	No	ASP-2	1	38
AY513728	cDNA	unknown	Yes	Yes	TS Trypo ligand	1	38
AY298908	gDNA	CLB	Yes	No	c71 surf protein	1	38
X70947	cDNA	CL	Yes	Yes	FL-160	1	3
X70948	gDNA	CL	Yes	No	FL-160-2	1	38
U59297	cDNA	Y	Yes	Yes	CRP-10	2	38
U01098	cDNA	Y	Yes	Yes	TS epi	0	0
X57235	gDNA	CAI	Yes	No	SAPA	0	0
AB188100	gDNA	Y	Yes	No	TS-193	0	0
D50685	gDNA	Y	Yes	No	TCTS-154	0	0

*Kozak consensus sequence [gccrccATGg; lower case r denotes a purine (adenine or guanine)], presence of predicted translation initiation site adjacent to the signal peptide, as determined by NetStart 1.0 Prediction Server.

**Spliced leader (SL), presence of a common 35-nucleotide sequence (SL) found at 5′-terminal part to the 5′ end of all trypanosome mRNAs.

***In-frame methionines. The numbers indicate the quantity of in-frame start codons (ATG) located up-stream from the one which lies adjacent to the signal peptide (SP). Internal SP sequences (after the first or second in-frame ATG) are predicted to be signal anchor sequences by SignalP 3.0 Server.

****Distance in amino acids (aa) from the furthest methionine up-stream from the one located adjacent to the signal peptide.

Ψ Pseudogene.

Furthermore, we found only one GP82 gene (XM_806823) with this structure in the genome of *T*. *cruzi* CLB. Additionally, in-frame start codons up-stream from the signal peptide is a feature that seems to be absent in the members (TS epi, SAPA, TS-193, TCTS-154) of TS superfamily that have *trans*-sialidase catalytic activity. We conclude that the presence of additional in-frame ATG starting codons in proteins deduced from genomic DNA data is not an artefact derived from the algorithm utilized to predict open reading frame, as this feature is also present in mRNA derived sequences as demonstrated by analysis of 28 cDNA clones shown in Table 1.

### The additional methionine in the N-terminal is not necessary for processing of GP82

**Figure 1:**
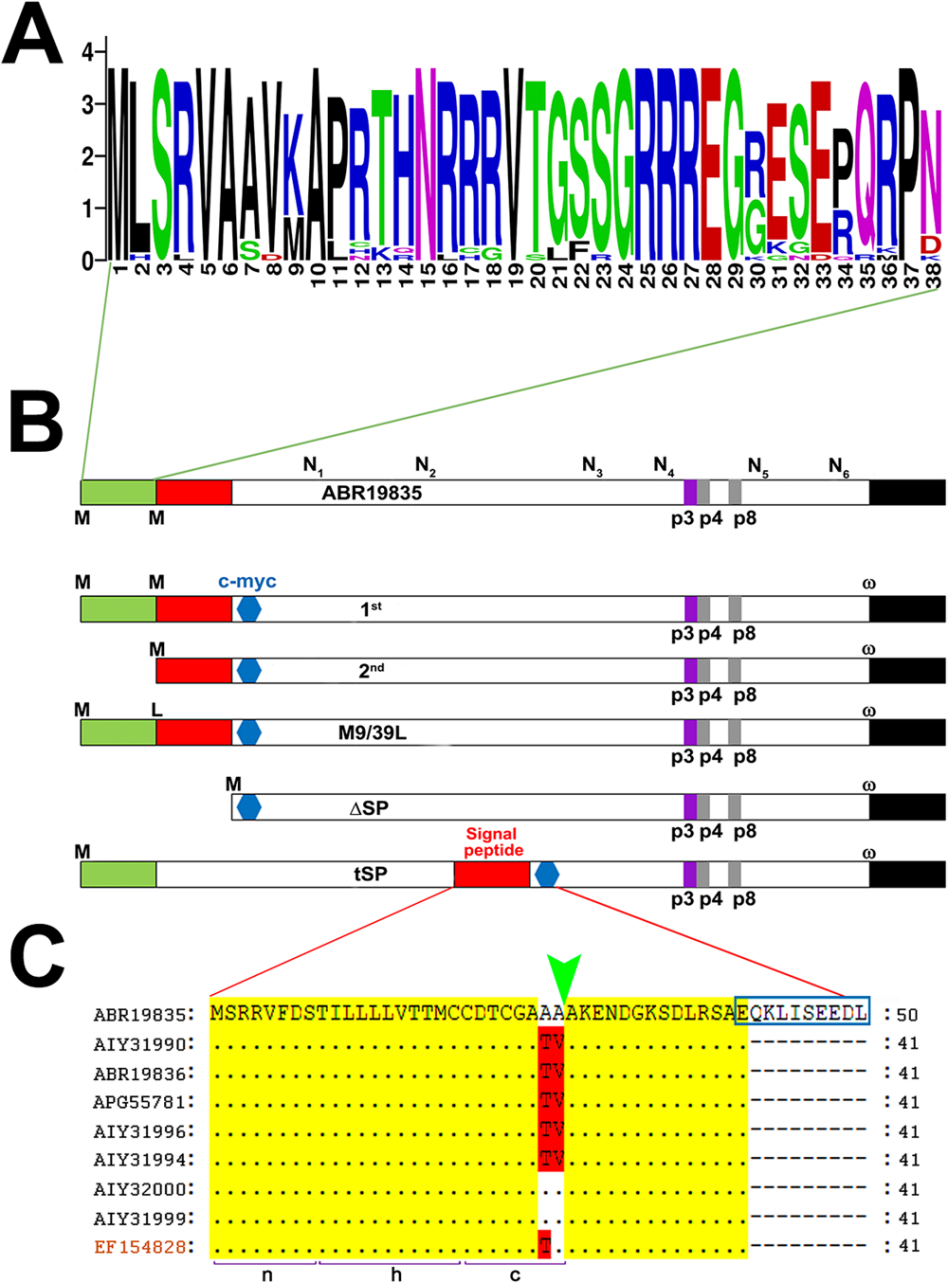
Schematic representation of GP82 constructs. (**A**) WebLogo representation of multiple sequence alignment (Clustal Omega) of uncharacterized 38 amino acids region from representative members of *trans*-sialidase family (from Table 1) indicating the relative frequency of amino acids at given position (height). (**B**) Illustrative representation of protein ABR19835 deduced from the cDNA 5.4G6 (GenBank EF154827) used as template to create the constructs 1^st^, 2^nd^, M9/39L, ΔSP and tSP. The N(_1–6_) depicted above the template protein, indicates putative *N*-glycosylation sites conserved throughout the constructs. In-frame methionines are indicated by the capital M letter (methionine at position 9 is not depicted). Capital L letter denotes the methionine to leucine substitution (M→L) introduced by site-directed mutagenesis (leucine at position 9 is not depicted) The green box denotes a 38 amino acids uncharacterized region between the 1^st^ and 2^nd^ constructs. The predicted SP is represented as a red box. The blue hexagon indicates the c-myc epitope introduced by PCR. P3, p4 and p8 denote the 3F6 monoclonal antibody epitope (p3), and the GP82 cell-binding sites (p4 and p8) involved in the interaction with the host-cell receptor. The black box symbolizes the GPI-anchor addition C-terminal signal where the ω is the GPI-anchor acceptor amino acid. (**C**) Alignment of SP of GP82 proteins derived from cDNA sequences (from Table 1) compared with c-myc tagged ABR19835 protein. Only the amino acids residues that differ from ABR19835 protein are identified, identical residues are depicted as dots. Protein accession numbers are indicated on the left. The green arrowhead denotes the predicted cleavage site by signal peptidase, between positions 27 and 28. The c-myc epitope (EQKLISEEDL) insertion site is indicated inside a blue box. Brackets labelled as n, h and c, denote the regions that compose the tripartite structure of GP82 signal peptide. EF154828 correspond to a GP82 pseudogene.

The N-terminal sequences of GP82 were aligned by Clustal Omega^38^ and the results exported into WebLogo^39^ for visualization. Noteworthy, the length (38 aa) and amino acid composition of the sequence located from the first putative methionine to the methionine adjacent to the SP are very conserved among GP82 proteins (Table 1; Fig. 1A). This stretch of 38 amino acids does not represent any known signal or functional domain, as determined by bioinformatics tools in the platforms Conserved Domain Database (CDD) and Domain Architectures (CDART)-NCBI, STRING, Interpro and Expasy. We envisioned that the presence of this small region could create three biosynthetic possibilities for these proteins, *i*.*e*.: (a) GP82 could be translated starting at the first available ATG start codon leaving the predicted SP as a signal anchor, (b) it could be translated immediately from the start codon adjacent to the SP as typical GPI-anchored proteins or (c) the protein could be translated from the first available start codon and then the SP removed by the signal peptidase. To further investigate the effect of those additional initiation codons in the SP processing, 3 constructs were engineered and cloned into pTEX plasmid for expression in transfected *T*. *cruzi* epimastigotes (non-infective developmental stage that does not express GP82 protein). The protein expression from this vector is driven by the intergenic regions of the constitutively expressed glyceraldehyde-3-phosphate dehydrogenase (GAPDH) protein, allowing transgene expression in all four developmental stages of *T*. *cruzi*^40^. Additionally, two constructs were engineered to characterize the SP and validate its functionality.

The Fig. 1B shows a scheme of the constructs generated in this study. Although some GP82 proteins present an in-frame methionine at position 9 (Fig. 1A), this is not a common trait among members of TS superfamily; therefore, we consider the methionine adjacent to the signal peptide (position 39) as the second (2^nd^) in-frame methionine. At the top, is shown the deduced protein ABR19835 encoded by the clone 5.4G6 (EF154827.2) isolated from a MT cDNA library^26,28^ showing the regions of interest. This is a full-length cDNA that has the spliced-leader (SL) sequence at 5′-UTR and poly A tail at 3′-UTR. The full translated protein shares 100% identity with the functional domains described from previous studies such as, mAb3F6 epitope, *N*-glycosylation sites, host-cell binding site and gastric mucin attachment site. As these domains/motifs determine the function of the native GP82 protein, the cDNA herein described is suitable to study the native protein and investigate previously uncharacterized regions. Furthermore, the GP82 (ABR19835) encoded by this cDNA is highly conserved in different *T*. *cruzi* strains. In order to distinguish the transgenic GP82 protein from the endogenous protein synthesized by MTs, the c-myc epitope was inserted into a region located 13 amino acids after the putative cleavage site of the signal peptidase (Fig. 1B,C). In this position, the epitope would remain attached to the mature protein in close proximity to its N-terminus, increasing its exposition and accessibility. The constructs pTEX-1^st^ and pTEX-2^nd^ were intended to investigate the processing of GP82 protein starting at two different positions. To rule out the translation from an internal start codon (2^nd^ methionine) in pTEX-1^st^ construct, we engineered the construct pTEX-M9/39L replacing the methionines (M) (positions 9 and 39) by leucine (L), using site-directed mutagenesis. In this construct, the translation was enforced to start at the first and furthermost start codon available (1^st^ methionine). The constructs pTEX-ΔSP and pTEX-tSP aimed to address the functionality of the SP and its behaviour as an internal signal, respectively. The protein encoded by the pTEX-ΔSP construct lacks the SP, and starts at an artificial ATG start codon inserted adjacent to the sequence encoding the amino acid found at the mature N-terminus after the signal peptidase cleavage. In the construct pTEX-tSP the SP was transposed to an internal region, while retaining the cleavage site and c-myc epitope.

**Figure 2:**
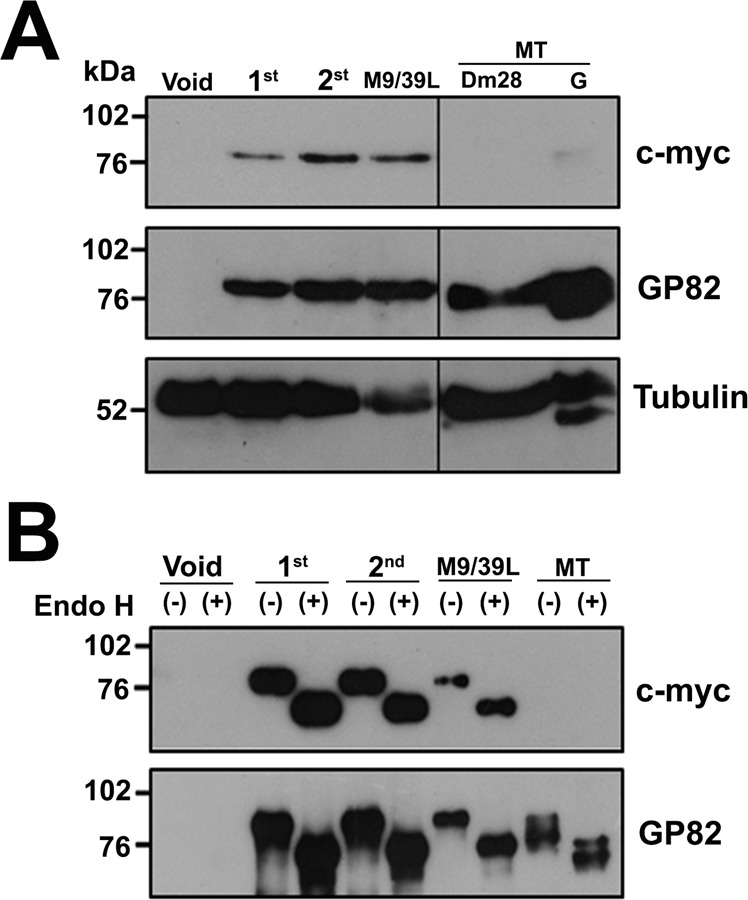
Expression of GP82 in transfected *T*. *cruzi* epimastigotes. (**A**) Three micrograms of proteins from total extracts of transfected epimastigotes were separated on 10% SDS-PAGE, transferred to nitrocellulose membranes and incubated with mAb 9E10 (anti-c-myc), mAb 3F6 (anti-GP82) or anti-tubulin monoclonal antibodies. Samples were washed and incubated with peroxidase conjugated antibodies and the immunocomplexes developed by chemiluminescence. Void: transfected epimastigotes carrying the empty pTEX vector; 1^st^: transfected parasites carrying the pTEX-1^st^ construct; 2^nd^: epimastigotes transfected with pTEX-2^nd^ construct; M9/39L: epimastigotes transfected with pTEX-M9/39L construct. MT: wild-type metacyclic trypomastigotes from clone Dm28c or G strain. The vertical black line inside the panels denotes the boundary between lanes from the same developed membrane that were non-adjacent in the gel. Protein molecular weight standards (kDa) are indicated on the left. (**B**) Endoglycosidase H digestion of GPI-anchored enriched-protein extracts from transfected *T*. *cruzi* epimastigotes. GPI-enriched samples from transfected epimastigotes (5 × 10^5^ parasite/equivalents) and MTs (1 × 10^5^ parasite/equivalents) were treated (+) or mock treated (−) with 750 U of endoglycosidase H (Endo H) at 37 °C for 3 h. Samples were separated on 10% SDS-PAGE, transferred to nitrocellulose membranes and incubated with anti-c-myc (upper panel) or anti-GP82 (lower panel) monoclonal antibodies. Immunocomplexes were developed as described in (**A**). Void: epimastigotes transfected with empty pTEX plasmid; 1^st^: transfected parasites carrying the pTEX-1^st^ construct; 2^nd^: epimastigotes transfected with pTEX-2^nd^ construct; M9/39L: transfected parasites carrying pTEX-M9/39L construct; MT: wild-type metacyclic trypomastigotes from clone Dm28c (1 × 10^5^ equivalent). Protein molecular weight standards (kDa) are indicated on the left. Full-length immunoblots are presented in Supplementary Fig. S5.

The expression of tagged-GP82 transcripts was assayed by immunoblotting in total or GPI-anchored protein enriched extracts from transfected epimastigotes, non-infective developmental stage that does not express GP82 protein. Figure 2A shows the expression profile of transgenic GP82 proteins in total epimastigote extracts incubated with monoclonal antibody (mAb) 9E10 specific for the c-myc epitope. The antibody mAb 9E10 reacted specifically with a single protein band of ~82 kDa, found only in epimastigotes transfected with the three GP82 constructs (Fig. 2A upper panel). No reactivity was observed in epimastigotes transfected with the void pTEX plasmid or in protein extracts from wild-type MTs, both lacking the c-myc epitope. This finding corroborates that the protein recognized by mAb 9E10 was not translated in epimastigotes from an endogenous GP82 gene. Membranes re-probed with mAb 3F6, which is specific for GP82^41^, showed the same bands detected by mAb 9E10 and revealed GP82 protein in extracts of wild type MTs but not in the population transfected with the empty plasmid (Fig. 2A middle panel). Stripped membranes re-probed with a mAb directed against the α-tubulin protein showed that equivalent amounts of protein were loaded in the gel (Fig. 2A lower panel). These results indicate that transgene protein expression was successfully accomplished for all constructs and the insertion of c-myc epitope did not impair GP82 processing.

No differences were observed in the electrophoretic mobility of transgenic GP82 proteins, suggesting that their processing at the N-terminus was similar. As stated above, there is a stretch of 38 aa from the first available methionine to the one that lies adjacent to the SP, with an expected molecular weight of 4.319 kDa. The predicted SP is 27 aa long (identified by SignalP 4.1 Server^42^) with an estimated molecular weight of 2.892 kDa. After ruling out the initiation of translation in an internal ATG start codon, the maximum difference expected for proteins with cleaved SP and those containing uncleaved signal anchor would be 7.211 kDa. Our own experimental data indicate that a 10% SDS-PAGE has enough resolution to differentiate the GP82 lacking one *N*-glycosylation site (see Supplementary Fig. S1), a post-translational modification that in *T*. *cruzi* accounts for ~1.8 kDa^43^. The aforementioned results indicate that the lack of differences in size observed in the transgenic proteins is not an artefact derived from the SDS-PAGE resolving power.

### Post-translational modifications (PTMs), processing and secretion of GP82 mutants

Native GP82 is a glycoprotein, containing *N*-linked oligosaccharides, attached to the cell membrane by a GPI-anchor^12,44^. To examine these post-translational modifications in GP82 mutants, we performed a protein extraction with detergent Triton X-114 (TX-114), which has proved to be a useful approach to produce fractions enriched in *T*. *cruzi* MT GPI-anchored proteins such as, GP82, GP90 and the 35/50 mucin complex^45^. The TX-114 detergent extracts of transfected parasites, enriched in GPI-anchored proteins, were treated with endoglycosidase H (Endo H), a glycosidase that cleaves high-mannose type *N*-linked oligosaccharides. Undigested detergent extracts and the endo H-treated samples were analysed by immunoblotting using mAb 9E10. GP82 and its *N*-deglycosylated counterpart were detected in all parasite populations carrying the GP82 transgene, but not in extracts enriched in GPI-anchored proteins from wild-type MTs or epimastigotes transfected with the void pTEX plasmid (Fig. 2B upper panel). Reaction with mAb 3F6 revealed GP82 and its *N*-deglycosylated counterpart in extracts from wild-type MTs and all transfected populations, excepting epimastigotes transfected with the void plasmid (Fig. 2B lower panel). No differences in the electrophoretic mobility of GPI-anchored transgenic GP82, as well as of proteins without *N*-glycosylation, were detectable among the different parasite constructs. The susceptibility of the transgenic GP82 to Endo H, similar to that observed for the wild-type MT GP82 protein (Fig. 2B lower panel), indicated a correct processing of tagged-proteins by *T*. *cruzi* oligosaccharyltransferase (OST). These results corroborate that GP82 transgenic proteins interacted with the ER-translocon complex and underwent posttranslational modification by addition of Endo H-sensitive *N*-linked oligosaccharides inside the ER. We also determined whether the GP82 encoded by episomal plasmids was secreted into the milieu, in the same fashion as previously described in MTs^46^. GP82 secretion was detected in parasites transfected with pTEX-M9/M39L construct, and to a lesser degree in parasites transfected with -2^nd^ construct, but not in those transfected with -1^st^ construct (Supplementary Fig. S2).

### SP is only functional as sorting signal when located at N-terminus of GP82

**Figure 3:**
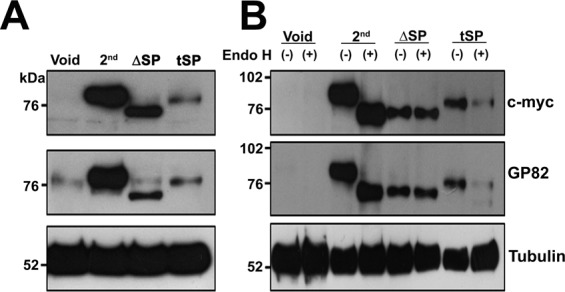
**(A) **SP alterations in GP82 influence its electrophoretic mobility. Total protein extracts from transfected epimastigotes (6 × 10^6^) were resolved on 10% SDS-PAGE, transferred to nitrocellulose membranes and incubated with 9E10 (anti-c-myc), 3F6 (anti-GP82) or anti-tubulin monoclonal antibodies. Void: epimastigotes transfected with empty pTEX plasmid; 2^nd^: transfected parasites carrying the pTEX-2^nd^ construct; ΔSP: epimastigotes transfected with the pTEX-ΔSP construct (without SP); tSP: transfected parasites carrying the pTEX-tSP construct (transposed SP). Protein molecular weight standards (kDa) are indicated on the left. (**B**) Modifications in the SP impacts GP82 glycosylation. Total protein extracts from transfected epimastigotes (6 × 10^6^) were treated (+) or mock treated (−) with 500 U of endoglycosidase H (Endo H) at 37 °C for 3 h. Samples were separated on 10% SDS-PAGE, transferred to nitrocellulose membranes and incubated with anti-c-myc (upper panel) or anti-GP82 (middle panel) monoclonal antibodies. Void: epimastigotes transfected with empty pTEX plasmid; 2^nd^: transfected parasites carrying the pTEX-2^nd^ construct; ΔSP: epimastigotes transfected with pTEX-ΔSP construct (without SP); tSP: transfected parasites carrying the pTEX-tSP construct (transposed SP). Protein molecular weight standards (kDa) are indicated on the left. Full-length immunoblots are presented in Supplementary Fig. S6.

SPs are required to address proteins to the ER lumen where they can be further modified by PTMs, associate with membranes or be secreted to the milieu. Figure 3A shows the expression of c-myc tagged constructs pTEX-2^nd^ and two variants: one lacking the SP (pTEX-ΔSP) and another carrying the SP in a transposed-internal position (pTEX-tSP). Immunoblotting of total extracts incubated with mAb 9E10 showed that proteins encoded by the aforementioned constructs were variable in size ranging from ~72 to 82 kDa. There was a ~10 kDa difference between the protein encoded by the pTEX-ΔSP construct and that encoded by pTEX-2^nd^. The expected difference between both constructs should be ~2.8 kDa as maximum, corresponding to the deletion of the 27 aa N-terminal SP. The large difference in size observed between these constructs strongly suggest that the first 27 amino acids of construct pTEX-2^nd^ were indeed a SP that drove the protein to the ER where it underwent PTMs (Fig. 3A). To further characterize the requirement of SP for protein PTM, proteins extracts from the above constructs were submitted to digestion with Endo H and analysed by immunoblotting. Distinct from the pTEX-2^nd^ encoded protein, which had its electrophoretic mobility increased after *N*-deglycosylation by Endo H, no difference in migration was observed for pTEX-ΔSP encoded protein after Endo H treatment (Fig. 3B), indicating that this protein did not undergo *N*-glycosylation, which was reinforced by its mobility, comparable to that of the deglycosylated form of the protein encoded by pTEX-2^nd^ construct. The expected size for the protein encoded by pTEX-ΔSP construct was 71.879 kDa (~72 kDa), a value that agrees with a previous finding showing that inhibition of *N*-glycosylation by treatment of MT with tunicamycin resulted in the expression of a ~70 kDa protein recognized by anti-gp82 mAb 3F6^44^. This result indicated that the construct pTEX-2^nd^, containing a 27 aa canonical SP, encoded the full version of GP82 and that the predicted SP is necessary to address the nascent GP82 to the ER, where it undergoes PTM such as *N*-glycosylation.

A prior study in complex eukaryotes demonstrated that signal peptidase was able to recognize and cleave the SP independent of its localization in the protein primary structure^47^. To examine if SPs from the early divergent eukaryote *T*. *cruzi* could be recognised independent of its localization, we transfected epimastigotes with the pTEX-tSP construct, expressing a GP82 in which the SP was transposed to an internal region (preceded by ~232 aa). Immunoblotting of protein extracts from transfected epimastigotes incubated with mAb 9E10 showed a slight difference in migration between proteins encoded by pTEX-2^nd^ and pTEX-tSP constructs (Fig. 3A,B). As the predicted size for the full-length pTEX-tSP construct was 78.896 kDa (~79 kDa), the detection of a corresponding protein band confirmed the translation from the first available methionine (Fig. 1A). The absence of bands of smaller size indicated that no proteins originated from cleavage by the signal peptidase. These results indicated that GP82 SP was not recognized nor cleaved, when located in an internal position away from the N-terminus. In order to investigate why the GP82 coded by pTEX-tSP construct was not cleaved by the signal peptidase, we analysed the association of this protein with the ER membrane. There are six putative *N*-glycosylation sites in the primary structure of GP82 constructs (Fig. 1A). After transposition of the SP (including the c-myc epitope tag), the *N*-glycosylation sequons distribute as follows: two sites before the SP and 4 sites located after the c-myc epitope (Fig. 1A). Endo H treatment of pTEX-tSP transfected epimastigote extract did not cause any alteration in GP82 electrophoretic mobility, suggesting that the protein encoded by this construct was neither embedded within the ER membrane nor gained access to the OST complex. Otherwise, it would be glycosylated, regardless of its topology (in-out or out-in). This result suggests that the internally placed SP did not function *per se* as a transmembrane domain and it was not able to drive ER transmembrane anchoring.

### Transgenic GP82 protein is expressed and functional at the surface of transfected epimastigotes

**Figure 4:**
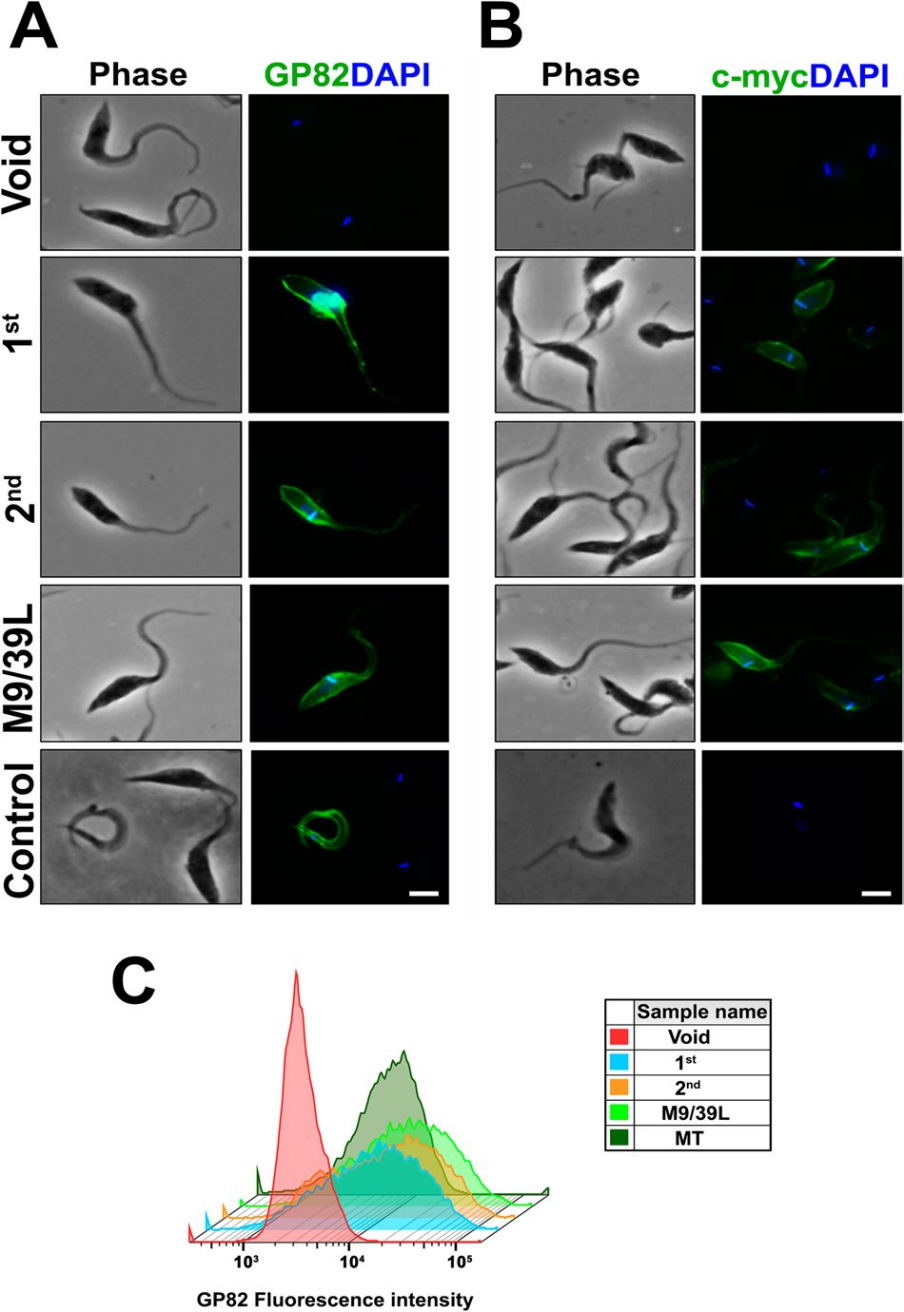
Immunofluorescence of transfected *T*. *cruzi* epimastigotes. (**A**) Live parasites were washed thrice with cold PBS and incubated with mAb 3F6 (anti-GP82) for 30 min on ice. Parasites were washed and incubated with 2 µg/mL Alexa Fluor 488 conjugated anti-mouse IgG antibody containing 200 nM DAPI. Samples were mounted using ProLong Gold antifade media and analysed on Olympus BX51 epifluorescence microscope. Void: epimastigotes transfected with empty pTEX plasmid; 1^st^: transfected parasites carrying the pTEX-1^st^ construct; 2^nd^: epimastigotes transfected pTEX-2^nd^ construct; M9/39L: transfected parasites carrying the pTEX-M9/39L construct; Control: metacyclic trypomastigotes from stationary phase cultures. (**B**) Fixed parasites were treated with methanol, blocked and incubated with mAb 9E10 (anti-c-myc) for 30 min on ice and processed as described above. Void: epimastigotes transfected with empty pTEX plasmid; 1^st^: transfected parasites carrying the pTEX-1^st^ construct; 2^nd^: epimastigotes transfected pTEX-2^nd^ construct; M9/39L: transfected parasites carrying the pTEX-M9/39L construct; Control: metacyclic trypomastigotes from stationary phase cultures. Bar: 3 μm. (**C**) Flow cytometry analysis of live transfected populations incubated with mAb 3F6. Live transfected epimastigotes and wild-type MT were washed and incubated with mAb 3F6 as described above. After final wash, samples were resuspended in PBS and their fluorescence levels analysed by flow cytometry on a BD Accuri C6 Flow Cytometer acquiring 10^4^ events.

The localization of tagged-GP82 expression was assayed in live parasites by immunofluorescence with mAb 3F6. The fluorescence signal was distributed evenly through the entire parasite surface, similar to that observed in MTs (Fig. 4A). This finding indicates that insertion of c-myc epitope did not impair GP82 folding and trafficking to the cell surface. No reactivity with mAb 9E10 was observed in live parasites, probably due to steric hindrance of the epitope. The reaction was also negative when PFA fixed parasites were used, probably due to epitope modification (masking). The decapeptide corresponding to the c-myc epitope contains a lysine residue at position 3 that could be modified by the amino-reactive cross-linker PFA. Although the amino acid at this position is not critical for antibody recognition and accept a broad range of substitutions in synthetic peptides^48^, it could be pivotal for epitope recognition under physiological conditions. When transfected parasites were fixed with 2.5% formaldehyde in PBS and dried onto slides, followed by treatment with methanol, the reaction with mAb 9E10 was positive, with a fluorescence profile characteristic of plasma membrane distribution (Fig. 4B). Flow cytometry analysis of live parasites incubated with mAb 3F6 confirmed the surface expression of GP82 transgene in the transfected populations. Transfected parasites showed fluorescence levels above the negative control carrying the void pTEX plasmid (Fig. 4C, Supplementary Fig. S3).

**Figure 5:**
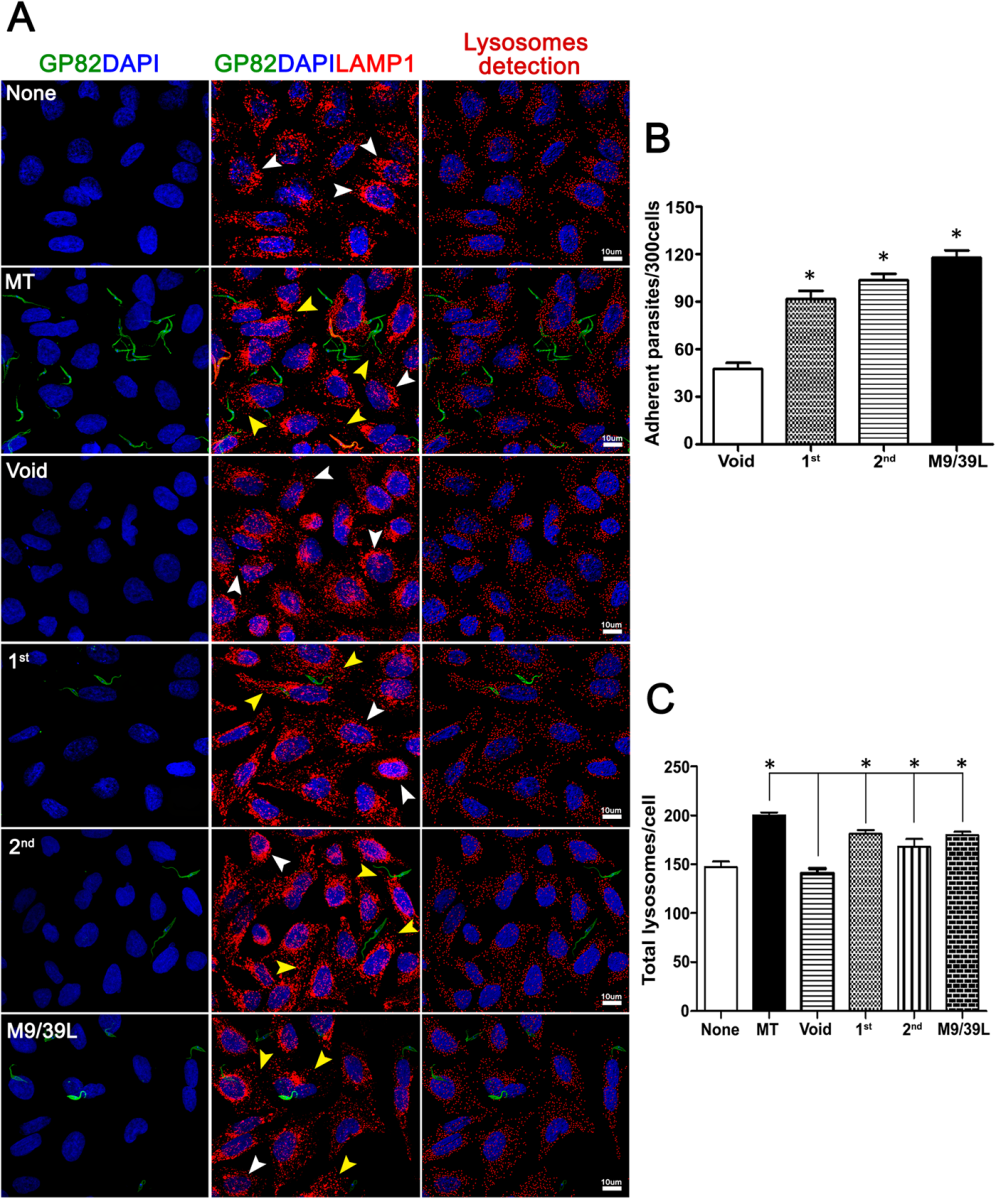
*T*. *cruzi* epimastigotes expressing GP82 bind to HeLa cells and induce lysosome mobilization. (**A**) HeLa cells seeded on glass coverslips were incubated with transfected parasites at MOI 20:1 for 1 h at 37 °C in 24-wells plates. Wells were washed with PBS to remove unbound parasites and were fixed with 4% PFA, permeabilized with 0.1% saponin (PGN-saponin) and incubated for 1 h with mouse mAb 3F6 and rabbit mAb anti-human Lamp1 diluted in PGN-saponin. Coverslips were washed and incubated for 1 h with 2 µg/mL of anti-mouse IgG conjugated to Alexa Fluor 488 and anti-rabbit IgG conjugated to Alexa Fluor 568 containing 1 µg/mL DAPI. Samples, mounted on microscopic slides using Prolong Gold, were analysed on Leica TCS SP8 Confocal Laser Scanning Platform using Leica Application suite (LAS) and Imaris (Bitplane) software packages. Lysosome detection was performed as described elsewhere^47^ None: cells incubated in culture media devoid of parasites; MT: wild-type metacyclic trypomastigotes; Void: epimastigotes transfected with empty pTEX plasmid; 1^st^: transfected parasites carrying the pTEX-1^st^ construct; 2^nd^: epimastigotes transfected pTEX-2^nd^ construct; M9/39L: transfected parasites carrying the pTEX-M9/39L construct. White arrowheads: perinuclear lysosomes. Yellow arrowheads: lysosome scattering induced by transfected parasites. Bar: 10 μm. (**B**) Epimastigotes expressing GP82 were incubated with HeLa cells at MOI 20:1 for 1 h at 37 °C in 24-wells plates containing DMEM medium. After washings with PBS to remove unbound parasites and fixation in Bouin solution followed by Giemsa staining, the coverslips were mounted onto microscopic slides and the number of cell-adherent parasites recorded by microscopy. The results correspond to the mean ± SD of parasites in 300 cells counted in triplicate (* p < 0.05). (**C**) Quantification of lysosome mobilization/scattering from panel A analysed by lysosome counting algorithm. Bars correspond to triplicates indicating mean ± SD of 10 different microscopic fields (≥300 cells) observed with a 63× objective (* p < 0.05).

To characterize the adhesive properties of GP82 transfected parasites we analysed their capacity to bind to HeLa cells. Epimastigotes expressing GP82 were capable to adhere to HeLa cells, similarly to MTs (Fig. 5A). Adhesion assay was also performed in nutrient-deprived medium (PBS^++^), which induces the lysosome scattering toward the cell surface (Supplementary Fig. S4), an event required for parasite internalization^49–51^. The number of adherent parasites, ranging 150–200 per 300 cells (Supplementary Fig. S4) was higher than in full nutrient medium that was on the order of 90–120 per 300 cells (Fig. 5B). The effect of transgenic GP82 on lysosome biogenesis and scattering was examined by immunofluorescence in HeLa cells incubated with GP82 transfected epimastigotes (Fig. 5A,C). Quantification of lysosomes revealed a significant increase in the total lysosome numbers in HeLa cells upon incubation with GP82 transfected epimastigotes (Fig. 5C). Despite their capacity of cell adhesion and lysosome biogenesis induction, epimastigotes expressing GP82 did not invade cells, indicating the requirement for additional molecules not present in this parasite stage. GP82 transfected epimastigotes were also tested for their ability to traverse the gastric mucin coat, by using Transwell filters coated with gastric mucin as described by^52^. Binding to gastric mucin is a critical step for MT migration through the mucus layer toward the gastric epithelial cells, which are the MT targets in oral *T*. *cruzi* infection^8^. GP82-transfected epimastigotes adhered to gastric mucin but were unable to pass through the gastric mucin-coated filter (data not shown), indicating that GP82 expression is not sufficient to confer the property to translocate through the gastric mucin coat.

## Discussion

A common structural feature of mRNAs encoding GPI-anchored *T*. *cruzi* TS proteins is the presence of one or two in-frame initiator codons up-stream from the canonical SP^21,26,28,32,53^. This region encodes a highly conserved 38 aa-peptide with an uncommon density of positively charged residues, suggesting a putative function that remains to be assigned. In the absence of other signals, the initiation of protein translation at an up-stream start codon implies that the SP became internal, acting as transmembrane domain (signal anchor). Since GPI-anchored TSs are released from the cell surface after treatment with phosphatidylinositol-specific phospholipase C^12^, we can rule out the transmembrane attachment at the protein N-terminus, although the translation starting at an up-stream start codon cannot be discarded. We herein investigated the functional role of the SP on the processing and membrane translocation of the GPI-anchored surface TS proteins using a full length GP82 cDNA (EF154827) as a model to generate GP82 mutants.

Our results strongly suggest that SP directs the translocation of GP82 across the ER membrane. First, epimastigotes transfected with the constructs pTEX-1^st^, -2^nd^ and -M9/39L expressed on the cell surface a protein of the same size of the native GP82. Given that construct pTEX-1^st^ carries the pTEX-2^nd^ start codon in a Kozak context, the initiation of translation at the second start codon in the pTEX-1^st^ construct cannot be discarded. To rule out this possibility, we created a third construct (pTEX-M9/39L) in which the start codon located immediately adjacent to the predicted SP and up-stream from it (residues 9 and 39 in the GP82-ABR19835), were mutated to encode the structural related amino acid leucine. Protein coded by this construct had an estimated molecular weight of ~82 kDa, similar to that coded by pTEX-1^st^ and -2^nd^ constructs, and also the wild-type MT, indicating that translation begins at first in-frame ATG codon and the nascent polypeptide chain is cleaved near the SP. The addition of *N*-linked oligosaccharides on GP82 mutant proteins and their expression on the parasite surface indicate a proper translocation and exposition to the OST active site at the ER lumen. We may suggest that ER translocating machinery of *T*. *cruzi* was able to recognize either the canonical SP in the pTEX-2^nd^ located immediately after the initiator codon or the signal anchor in the pTEX-1^st^ located 38 aa after the first initiator codon. This later result refutes previous statements suggesting that translation only initiates at the start codon encoding the SP^21,53^.

Second, no cleavage was observed when SP was located internally at ~232 aa from the GP82 N-terminus (pTEX-tSP), indicating that transposition of SP to an internal region rendered the signal unrecognizable by the signal peptidase. This lack of cleavage could be due to an improper exposition of the SP into the ER lumen or to incapacity of GP82 protein to associate with the ER membrane. The absence of *N*-linked oligosaccharides in this protein confirms that it was not addressed to the ER. Our data suggest that transposed SP did not become embedded within the ER membrane, supporting that the absence of cleavage was due to the fact that the GP82 SP lacks the proper hydrophobicity to function as a transmembrane domain. Additionally, the expression of a ~79 kDa GP82 by the construct pTEX-tSP confirmed that the translation initiated at the first available start codon, reinforcing the feasibility of wild type translation products conveying the 38 aa amino-terminal stretch such as those encoded by pTEX-1^st^ and pTEX-M9/39L constructs. These findings agree with previous observations that the SP of haemagglutinin was not cleaved at an internal position when preceded by a 111 amino acids^54^. In this model the cleavage of the haemagglutinin SP was only observed when the signal was preceded by 11 amino acids. Similar to the pTEX-tSP, the 111 aa internal haemagglutinin SP was incapable to direct the polypeptide for association with the ER membrane^54^. Conversely, mammalian ER translocation machinery was able to efficiently process an engineered protein containing a 109 aa-peptide extension preceding the SP of preprolactin^55^. In this model, the internal SP drove the translocation of the complete chimera into the lumen of ER where the signal peptidase cleaved the SP^55^. This finding suggests that recognition by the translocon and signal peptidase is influenced by the nature of the SP and probably the composition of the flanking protein sequence. Additional experiments are due to address the ability of *T*. *cruzi* signal peptidase to recognize an internally placed SP presented in another context, *e*.*g*., as part of a polytopic protein^47^.

Third, deletion of the SP (pTEX-ΔSP), produced a protein devoid of *N*-linked oligosaccharides with a molecular weight indistinguishable from the GP82 precursor^44^. This finding corroborated that the 27 aa deleted sequence corresponds indeed to a canonical signal peptide and additionally allowed to identify the minimal precursor capable to produce the full-length GP82 (*i*.*e*., pTEX-2^nd^). No immunofluorescence signal was detected in this transfected population (not shown), due to a low level of transgene expression. Although, no localization data was obtained for this construct, we would suggest that the protein lacking the signal peptide would distribute discretely in the epimastigotes cytoplasm, analogous to that observed by Canepa *et al*.,^56^ with a SP-truncated mucin construct. Amino terminal sequencing of purified mature transgenes is due to experimentally confirm the exact length and the cleavage site of the GP82 signal peptide herein unveiled.

Taken together, these results strongly suggest that the signal peptide determines its own cleavage inside the protein, in a process that shows strict dependence on the signal location. To the best of our knowledge, our findings represent the first report of an internal signal peptide in an early divergent protozoan parasite. Additionally, the detection of the transgene product pTEX-tSP at ~79 kDa indicated that, in this construct, the translation initiated at the first available methionine, reinforcing the feasibility of wild type translation products conveying the 38 aa amino-terminal stretch such as those encoded by pTEX-1^st^ and pTEX-M9/39L constructs.

We have shown that deletion of SP sequence or its translocation to an internal region blocks the biogenesis of GP82. Cleavage of SP is critical for glycosylation and addition of a GPI anchor into GP82, and to the translocation of mature protein to the cell surface. After extraction of transfected epimastigotes with TX-114, GP82 recombinant proteins were recovered in the detergent phase suggesting that they were linked to cell surface by a GPI anchor. We also demonstrated that GP82 proteins were secreted into the milieu, in the same fashion as previously described in MTs^46^. These findings offer additional experimental evidence that recombinant GP82 protein expressed by transfected epimastigotes underwent the same processing as the native protein expressed by MTs and followed a secretion pathway analogous to that present in MTs.

GP82 protein encoded by constructs pTEX-1^st^, -2^nd^ and -M9/39L were properly processed to the cell surface, leaving its functional domains such as, p3, p4, p8 and p7^8,57,58^ exposed on the outer leaflet. GP82-transfected epimastigotes adhered to HeLa cells inducing lysosome scattering toward the cell surface and also bound to gastric mucin, similarly to MTs^49,50,59^. However, transfected epimastigotes were unable to being internalized into HeLa cells or to pass through gastric mucin-coated filters, reinforcing the notion that the surface molecule GP82 is required but not sufficient for parasite internalization^59,60^.

Our results demonstrate that translocation of GP82 into the ER membrane involves a cleavable N-terminal SP in the precursor protein followed by *N*-glycosylation and cleavage/addition of a GPI anchor at C-terminus. In *T*. *brucei*, SP-containing proteins can be translocated to the ER by co- or post-translational pathways. However, only GPI anchored surface proteins are transported exclusively through the post-translational protein translocation^14–16,61^. Homologues of proteins involved in the transport of SP-containing proteins into the ER have also been identified in *T*. *cruzi*^14,61^, but they were not yet experimentally validated in this parasite. By analogy with *T*. *brucei* we could assume that the GP82 would be transported, post-translationally, by an SRP-independent pathway and the SP cleaved by the signal peptidase complex.

## Methods

### Parasites

The clone Dm28c of *T*. *cruzi* Dm28 strain isolated from *Didelphis marsupialis* was used in this study^62^. Epimastigotes forms were obtained from exponential growth phase cultures in LIT medium containing 10% of foetal bovine serum (FBS) at 26 °C. MTs were purified from stationary phase cultures (10–14 days) by anion exchange chromatography using DEAE-cellulose columns^41^.

### Multiple sequence alignment

Protein sequences representing the 38 aa region encoded by representative members of trans-sialidase family were aligned using the web based program Clustal Omega^38^ under default parameters. Results were exported into WebLogo^39^ application to generate a graphical representation of the multiple sequence alignment.

### GP82 c-myc epitope tagging

The insertion of c-myc epitope (EQKLISEEDL) into the GP82 coding sequence was performed by overlapping PCR^63,64^ using *Pfu* DNA polymerase (Fermentas). The cDNA clone 5.4G6 (GenBank EF154827) isolated from a MT library cloned into the pCMV-SPORT6 vector^26^ was used as template in two separated PCR reactions. Amplifications were performed using the oligonucleotides 4G6stF and cmyNR or the cmyNF and 82p4R oligonucleotides (see Supplementary Table S1 for oligonucleotides used in this study). Amplicons were resolved on agarose gel, purified and combined in equimolar quantities in a third PCR mixture. The reaction proceeded for 5 cycles in absence of oligonucleotides, followed by addition of 4G6stF and 82p4R oligonucleotides and proceeding for additional 30 cycles. Amplicons were purified, A-tailed and cloned into pGEM-T Easy Vector (Promega) for sequencing. Clones with the correct sequence were digested with BamHI and EcoNI restriction endonucleases and the purified insert sub-cloned into the 5.4G6 clone digested with the referred enzymes. The resulting clone named 5.4G6 c-myc was additionally amplified with the oligonucleotides senses 4G6stF or 4G6ndF and 82MatBF containing the XbaI and BamHI restriction sites, respectively and the P82HinR antisense oligonucleotide, containing the HindIII restriction site. The resulting PCR products were cloned into pTEX vector^38^ digested with the same enzymes generating the construct pTEX-1^st^, pTEX-2^nd^ and pTEX-ΔSP. A construct containing the SP in a transposed position was prepared by sequence overlap PCR using 8 oligonucleotides, as described above (oligonucleotides 1, 6 and 12–17 from Supplementary Table S1). The PCR mixtures included the pTEX-1^st^ construct as template and the resulting amplicons were cloned for sequencing and sub-cloned into pTEX vector as aforementioned.

### Site-directed mutagenesis

To eliminate the in-frame methionines located adjacent to the SP and upstream from it, a site-directed mutagenesis was performed by overlapping PCR^63,64^ using the 5.4G6 c-myc clone as template. The product of a PCR using the MtoLF and M2toLR primers was combined with the amplicon resulting from a PCR using the M2toLF and 82p4R oligonucleotides. The mixture cycled in absence of primers (5 cycles) was followed by addition of 1stMtoLXbF and 82p4R oligonucleotides. Amplicons were A-tailed, cloned into pGEM-T Easy Vector and sequenced to confirm identity. The clones were digested with XbaI and EcoRI restriction enzymes and the insert combined with an EcoRI/HindIII fragment encoding the C-terminal portion of GP82 molecule isolated from pTEX-1^st^ construct. The ligated fragments were cloned into pTEX vector digested with the XbaI and HindIII enzymes creating the construct pTEX-M9/39L.

### Parasite transfection

Transfections were performed using the Amaxa 4D-Nucleofector (Lonza) and supplemented with P3 primary cell solution, following the manufacturer’s recommendations. Briefly, epimastigotes from exponential growth phase cultures were harvested by centrifugation (5 min at 3,000 × *g*) and the cell pellet adjusted to 4 × 10^8^ parasites/mL in supplemented P3 primary cell solution. Cell suspensions (100 µL) were transferred to nucleocuvettes containing 10 µg of plasmid DNA and submitted to “Nucleofection” using the EH-100 protocol. The cuvette contents were transferred to 5 mL of LIT medium and incubated at 26 °C for 48 h before adding 200 µg/mL of G-418. Parasite populations showing resistant phenotype were analysed after 3 months of selection.

### Indirect immunofluorescence and flow cytometry

Epimastigotes in exponential growth phase were washed thrice with PBS and adjusted to 4 × 10^7^ parasites/100 µL of PBS containing 5% of FBS. Suspensions containing live parasites were incubated on ice with 3F6 monoclonal antibody^41^ for 30 min. Cells were washed with cold PBS and fixed with PBS containing 3.5% formaldehyde for 10 min on ice. Free aldehyde groups were quenched with PBS containing 30 mM Tris-HCl pH 8.0 and the samples deposited onto 12 wells slides and air dried. Slides were blocked for 30 min with PBS-5% FBS and incubated with 2 µg/mL of anti-mouse IgG conjugated to Alexa Fluor 488 (Molecular Probes) and 200 nM DAPI. Slides were washed with PBS, air-dried and mounted using ProLong Gold antifade reagent (Life Technologies). Slides were analysed in Olympus BX51 fluorescence microscope using a 100×/1.30 oil immersion objective and the images acquired with Olympus DP71 digital camera. Images were processed using Image Pro 6.0 Analysis Software (Media Cybernetics, Inc.). To detect the c-myc epitope tag, epimastigotes in exponential growth phase were washed thrice with PBS and fixed as described above. Samples were deposited onto 12-well microscope slides, air dried and incubated with −20 °C methanol for 10 min. Samples were blocked with PBS-5% FBS for 10 min and incubated with 9E10 monoclonal antibody (Sigma) for 30 min. Slides were washed with PBS and incubated with 2 µg/mL of anti-mouse IgG conjugated to Alexa Fluor 488 (Molecular Probes) and 200 nM DAPI. Slides were washed, mounted and visualized as described above.

Flow cytometry analysis was performed basically as described above. Briefly, parasites (4 × 10^7^) were washed with cold PBS, incubated on ice-bath with mAb 3F6 for 30 min, followed by incubation with anti-mouse IgG conjugated to Alexa Fluor 488. After washes in cold PBS and fixation with PBS containing 3.5% formaldehyde, the parasites were washed and resuspended in PBS for analysis in BD Accuri C6 Flow Cytometer (BD Biosciences). A total of 10^4^ events were acquired per sample and the data was exported as FCS files for analysis in FlowJo v10.1 software (FLOWJO, LLC).

### Parasite adhesion to HeLa cells and lysosome mobilization assays

HeLa cells (1.5 × 10^5^) were seeded onto 13-mm diameter glass coverslips in 24-well plates and incubated at 37 °C in a 5% CO_2_ humidified atmosphere. Cells were incubated with transfected epimastigotes at MOI 20:1 for 1 h at 37 °C, washed with PBS to remove unbound parasites and fixed in Bouin solution followed by Giemsa staining. Coverslips were mounted onto microscope slides and the number of cell-adherent parasites recorded in triplicate by counting 300 cells. Significance levels were determined by Student t-test, comparing the amounts of parasites adhered to HeLa cells. Lysosome mobilization assay was performed as described elsewhere^49^ with minor modifications. HeLa cells were incubated with parasites for 1 h. Following the removal of unbound parasites, the samples were fixed with 4% PFA for 30 min and quenched with 50 mM NH_4_Cl. The cells were permeabilized for 10 min with PBS containing 0.15% gelatin, 0.1% sodium azide and 0.1% saponin (PGN-saponin) and incubated for 1 h with mouse mAb 3F6 and rabbit mAb anti-human lysosome-associated membrane protein 1 (Lamp-1, Cell Signaling Technology) diluted in PGN-saponin. Coverslips were washed and incubated for 1 h with 2 µg/mL of anti-mouse IgG conjugated to Alexa Fluor 488 and 2 µg/mL of anti-rabbit IgG conjugated to Alexa Fluor 568 containing 10 µg/mL DAPI. Samples were washed and mounted onto microscope slides using ProLong Gold antifade reagent. Images were acquired in Leica TCS SP8 Confocal Laser Scanning Platform (Leica, Germany) at Instituto de Farmacologia e Biologia Molecular (INFAR), Universidade Federal de São Paulo. Images were analysed using Leica Application suite (LAS), Imaris (Bitplane) and Adobe Photoshop CS6 (Adobe Systems Inc.) software packages. Significance was determined by Student t-test (GraphPad Software, Inc), comparing the values of lysosome mobilization induced by each transfected population and the basal values obtained in cells incubated in culture medium.

### GPI-anchored protein extracts

Enrichment of GPI-anchored proteins was performed as described by Cordero *et al*.^45^. Briefly, parasites were washed thrice with PBS and lysed on ice in a solution containing 10 mM Tris-HCl, 150 mM NaCl containing 2% of TX-114 (TBS-2% TX-114) supplemented with complete Mini EDTA-free Protease Inhibitor Cocktail Tablets (Roche) and 1 mM PMSF. Samples were clarified at 8,800 × *g* and the supernatant incubated at −20 °C for 24 h. Samples were thawed and submitted to 4 consecutive temperature-induced phase separations at 37 °C. Samples were clarified at 18,000 × *g* for 10 min at 0 °C and submitted to a final phase separation at 37 °C. The final detergent-rich phase was precipitated with 3 volumes of cold acetone and the proteins recovered by centrifugation at 18,000 × *g*. Aliquots were analysed by SDS-PAGE stained with colloidal Coomassie or silver nitrate and by immunoblotting after transfer onto nitrocellulose membranes.

### Deglycosylation of protein extract

Proteins extracts enriched in GPI-anchored proteins were resuspended in 10 µL of a solution containing 0.5% SDS and 40 mM DTT and denatured for 10 min at 100 °C. Samples were allowed to cool and the reaction volume completed to 20 µL in 50 mM of sodium citrate pH 5.5 containing 500 U of endoglycosidase H (Endo H, New England Biolabs). The reactions were carried out at 37 °C for 3 h followed by inactivation at 75 °C for 10 min. Samples were resolved on 10% SDS-PAGE and the extent of deglycosylation analysed by immunoblotting using 3F6 and 9E10 monoclonal antibodies. For deglycosylation of total parasite extracts, epimastigotes were washed thrice with PBS and adjusted to a concentration of 1 × 10^8^ parasites/mL in PBS supplemented with complete Mini EDTA-free Protease Inhibitor Cocktail Tablets (Roche) and 1 mM PMSF. Aliquots containing 5 × 10^6^ cells were frozen and concentrated to dryness in SpeedVac (Heto). Samples were resuspended and processed as aforementioned.

## Supplementary information

Supplementary information
