# Supplementary material for: Signal peptide recognition in Trypanosoma cruzi GP82 adhesin relies on its localization at protein N-terminus
Source: Sci Rep. 2019 May 13;9:7325. doi: 10.1038/s41598-019-43743-0 (PMC6513831; doi:10.1038/s41598-019-43743-0)
Supplement: Supplementary file 1 — Supplementary information [file 41598_2019_43743_MOESM1_ESM.pdf]

Signal peptide recognition in *Trypanosoma cruzi* GP82 adhesin relies on its location at protein *N*-terminus

Esteban M. Cordero[1, 2], Cristian Cortez[1, 2], Nobuko Yoshida[2] & José Franco da Silveira[2, *]

[1] Departamento de Microbiologia, Imunologia e Parasitologia, Escola Paulista de Medicina-Universidade Federal de São Paulo (UNIFESP-EPM), Brasil.
[2] Centro de Genómica y Bioinformática, Facultad de Ciencias, Universidad Mayor, Santiago, Chile.

SUPPLEMENTARY INFORMATION

TABLE OF CONTENTS

Supplementary Figures

Figure S1	GP82 transgene expression by transfected epimastigotes	S-2
Figure S2	Secretion of transgenic GP82 by transfected epimastigotes	S-3
Figure S3	Surface expression of GP82 transgene on transfected epimastigotes	S-4
Figure S4	Adhesion of transfected epimastigotes expressing GP82 constructs onto HeLa cells	S-5
Figure S5	Full-length immunoblots from figure 2	S-6
Figure S6	Full-length immunoblots from figure 3	S-7

Supplementary Table

Table S1	Oligonucleotides used in this study	S-8

Miscellaneous

S1	Deduced amino acid sequences of GP82 constructs generated in this study	S-9

References S-11

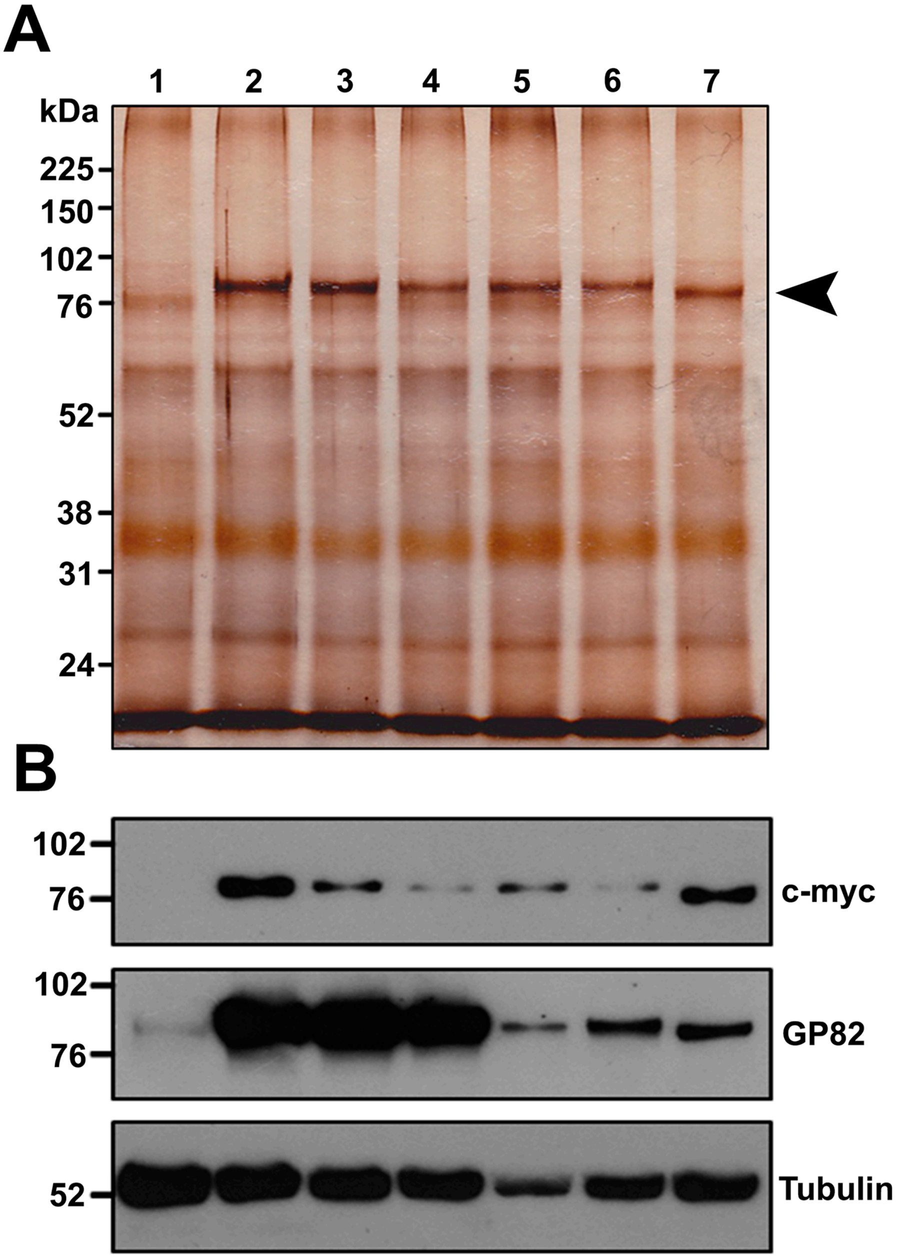

Figure S1. GP82 transgene expression in transfected epimastigotes. Protein extracts enriched in GPI-anchored proteins (A) or total extracts (B) from parasites transfected with several GP82 constructs were resolved in 10 % SDS-PAGE and protein bands visualized by silver stain (A) or transferred onto nitrocellulose membranes (B). Membranes were incubated with mAbs specific for the indicated proteins and the immunocomplexes developed by chemiluminescence. Molecular weight standards are indicated on the left in kDa. Numbers on the top indicate the different GP82 constructs transfected into *T. cruzi* epimastigotes: 1, pTEX-Void construct; 2, pTEX-1st construct; 3, pTEX-2nd construct; 4, pTEX-M9/39L construct; 5, pTEX-M9/39LF construct (same as number 4, but included a C-terminal FLAG epitope located before the GPI-anchor addition signal); 6, pTEX-1stF construct (same as number 2, but included a C-terminal FLAG epitope located before the GPI-anchor addition signal); 7, pTEX-1stN573Q construct (same as number 6, but included a mutation in the *N*-glycosylation sequon located at position 573 replacing the amino acid asparagine by glutamine). The black arrowhead indicates the shift in electrophoretic mobility corresponding to the lack of one *N*-glycosylation site.

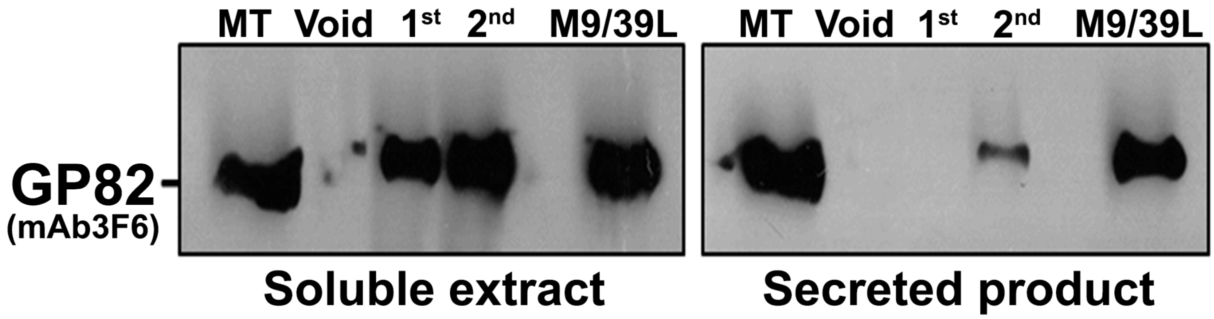

Figure S2. Secretion of transgenic GP82 by transfected epimastigotes. Transfected epimastigotes were washed and adjusted to 1×10^9 parasites/mL in Dulbecco's modified Eagle's medium supplemented with 10% foetal bovine serum (D10) and incubated at 37 °C for 1 h, as described elsewhere[1]. Samples were centrifuged and protein extracts equivalent to 1×10^7 parasites from pellet (soluble extract) and supernatant (secreted product) were resolved by SDS-PAGE and analysed by immunoblotting with mAb 3F6. MT: wild type metacyclic trypomastigotes; Void: pTEX-Void construct; 1st: pTEX-1st construct; 2nd: pTEX-2nd construct; M9/39L: pTEX-M9/39L construct.

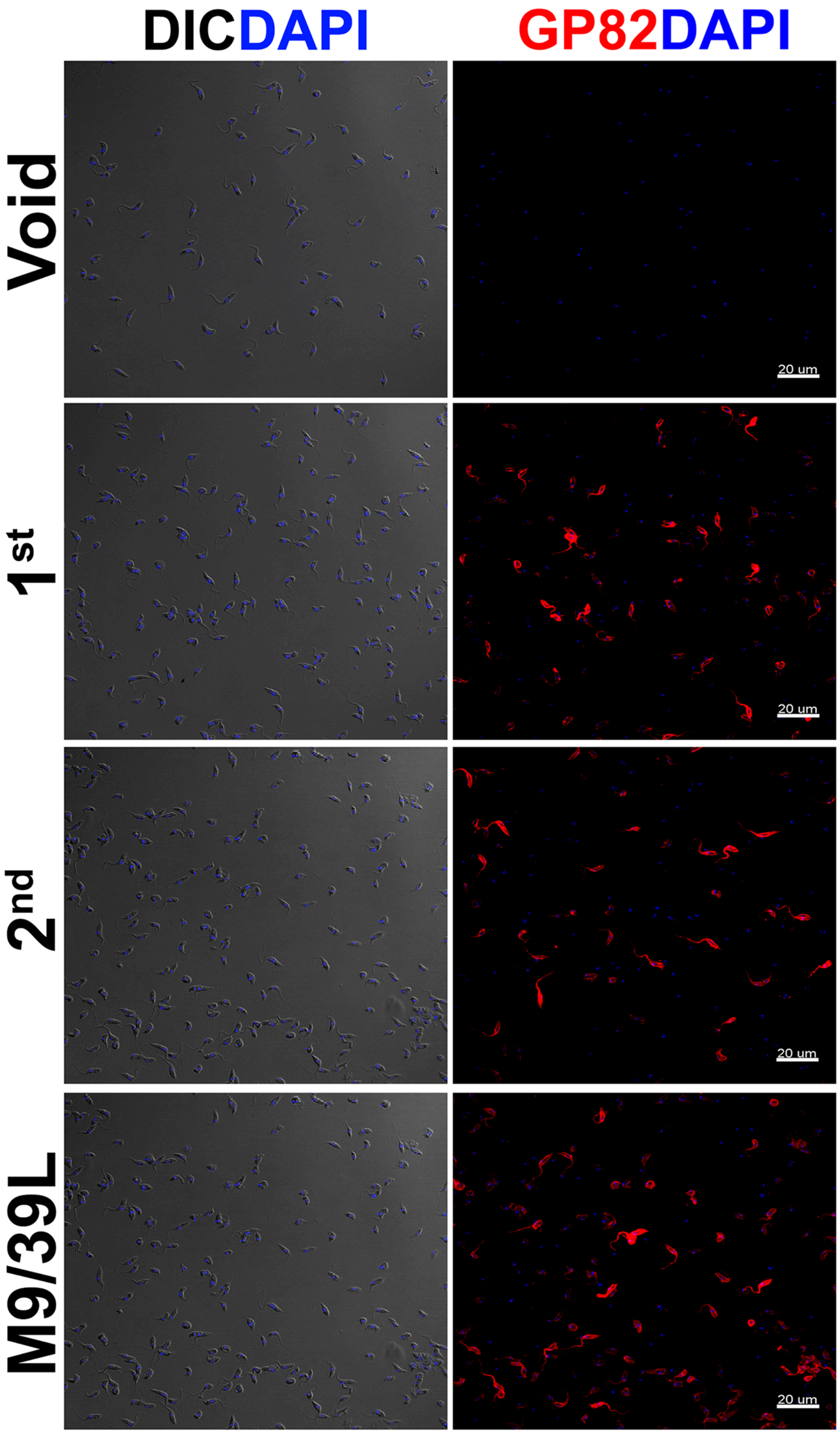

Figure S3. Surface expression of GP82 transgene on transfected epimastigotes. Live transfected epimastigotes were washed and incubated on ice with mAb 3F6 followed by incubation with Alexa-488 conjugated anti-mouse IgG. Samples were fixed and mounted onto microscope slides using ProLong Gold antifade media. DNA rich structures were stained with DAPI. Images were acquired in Leica TSC SP8 laser scanning confocal microscope using a 63× oil immersion objective and assembled using Imaris package. Void: pTEX-Void construct; 1st: pTEX-1st construct; 2nd: pTEX-2nd construct; M9/39L: pTEX-M9/39L construct. DIC: differential interference contrast, Blue: DNA rich structures stained with DAPI; Red: GP82 protein pseudo-coloured (Alexa-488 emission). Bar: 20 μm.

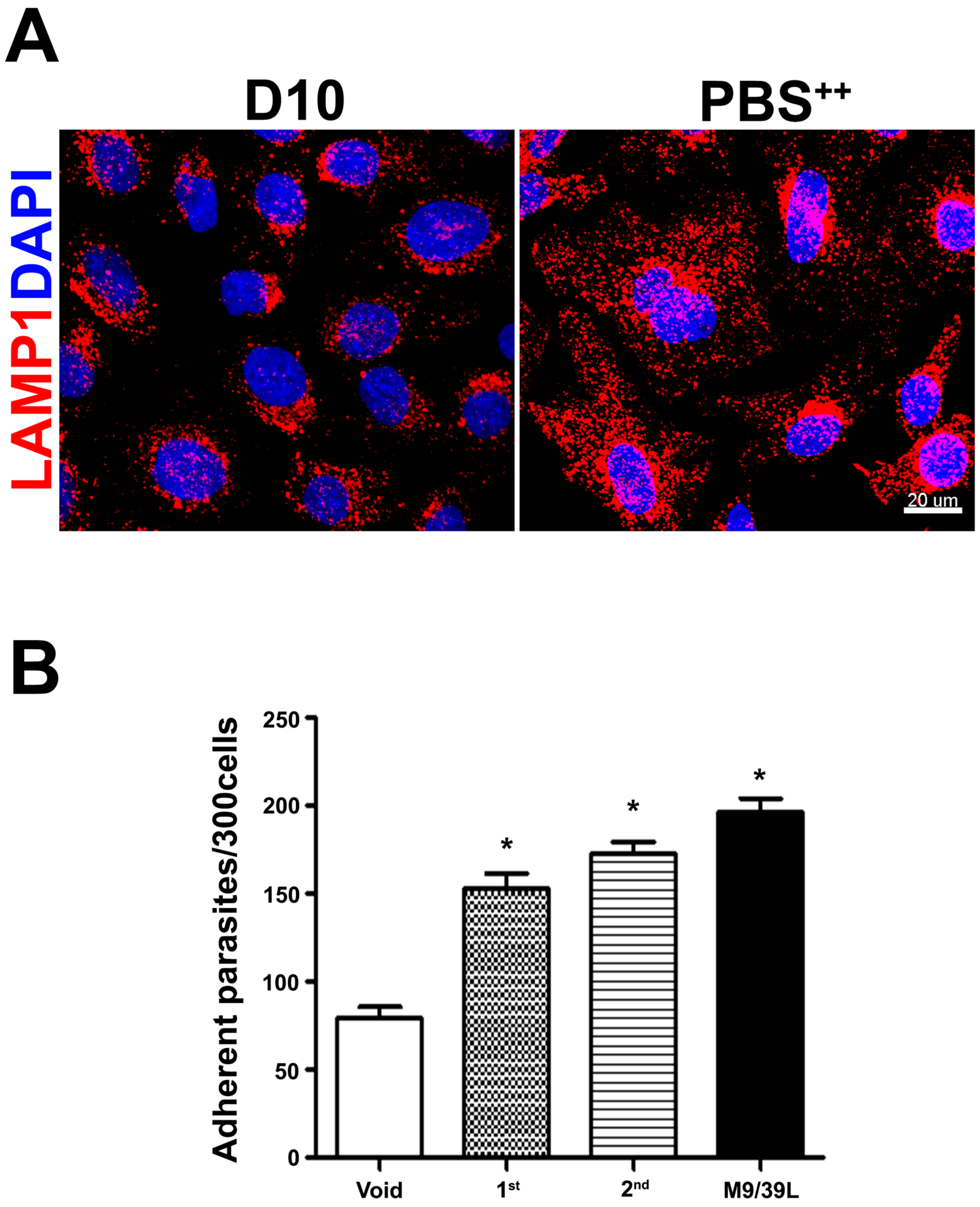

Figure S4. Adhesion of transfected epimastigotes expressing GP82 onto HeLa cells. **A)** Immunofluorescence (with anti-Lamp-1 antibody) of HeLa cells incubated in normal culture medium (D10) or in nutrient-deprived medium (PBS^{++}). Blue: DNA rich structures stained with DAPI; Red: lysosomal protein Lamp-1 protein pseudo-coloured (Alexa-488 emission). Bar: 20 µm. **B)** Adhesion of GP82 transfected parasites to the surface of HeLa cells assayed in nutrient-deprived medium. The assay was performed as described in "experimental procedures section". Void: pTEX-Void construct; 1st: pTEX-1st construct; 2nd: pTEX-2nd construct; M9/39L: pTEX-M9/39L construct.

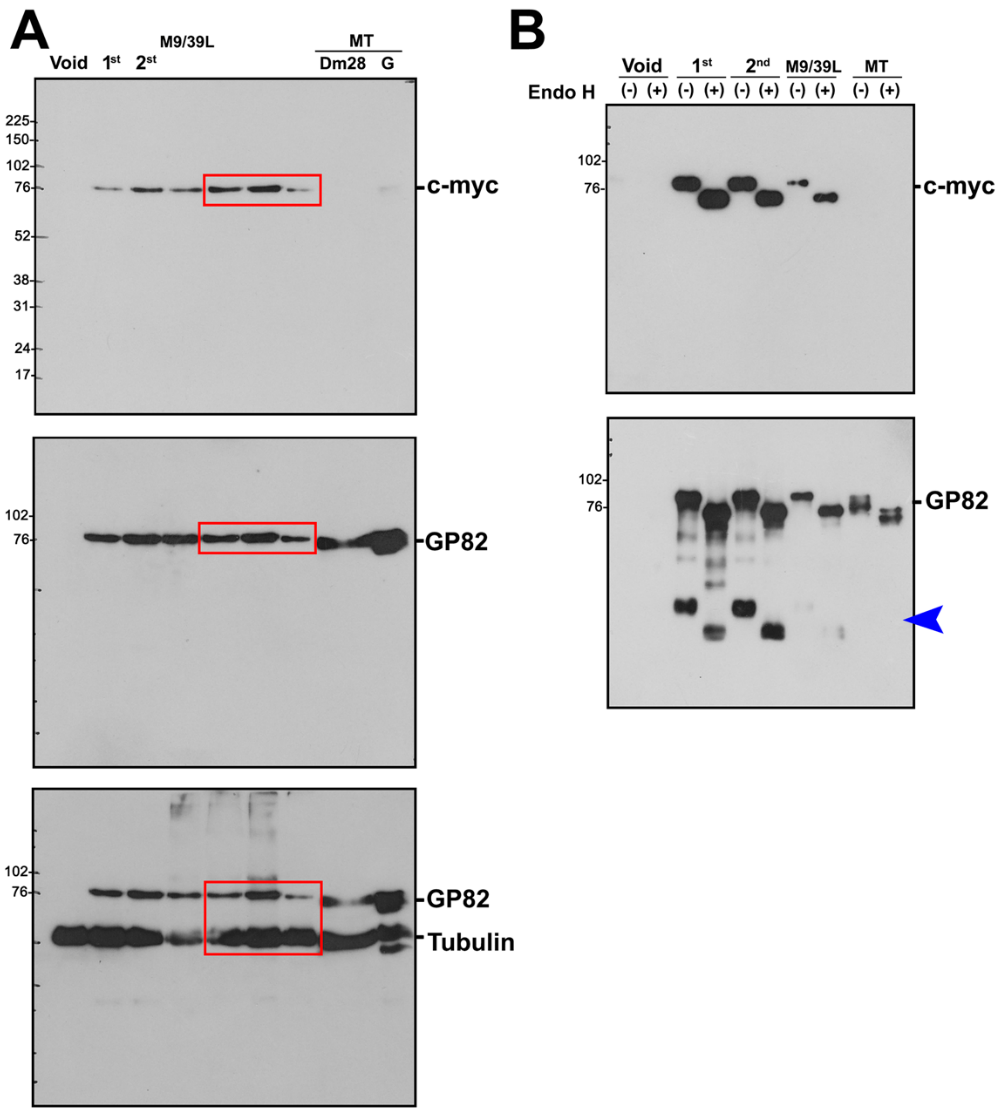

Figure S5. Full-length immunoblots from figure 2. A) Three micrograms of proteins from total extracts of transfected *T. cruzi* epimastigotes were separated on 10% SDS-PAGE, transferred to nitrocellulose membranes and incubated with mAb 9E10 (anti-c-myc). Samples were washed and incubated with peroxidase conjugated antibodies and the immunocomplexes developed by chemiluminescence. Membranes were stripped and incubated with mAb 3F6 (anti-GP82) followed by anti-tubulin monoclonal antibodies and developed as described. Void: transfected epimastigotes carrying the empty pTEX vector; 1st: transfected parasites carrying the pTEX-1st construct; 2nd: epimastigotes transfected with pTEX-2nd construct; M9/39L: epimastigotes transfected with pTEX-M9/39L construct. MT: wild-type metacyclic trypomastigotes from clone Dm28c or G strain. Red boxes denote the samples absent in figure 2 in order to display lines that were non-adjacent in the gel. **B)** GPI-enriched samples from transfected epimastigotes (5 × 10^5 parasite/equivalents) and MTs (1 × 10^5 parasite/equivalents) were treated (+) or mock treated (-) with 750 U of endoglycosidase H (Endo H) at 37 °C for 3 h and analysed by immunoblotting with 9E10 (upper panel) or 3F6 (lower panel) monoclonal antibodies. The blue arrowhead indicates an additional ~30 kDa 3F6-reactive protein found in total extracts of some *T. cruzi* stains, such as CL[2] and Y[3] and GPI-anchored enriched-protein extracts from G strain[4]. This GP82-related protein is also present in some strains lacking GP82 expression[2]. Sample labels: Void, 1st, 2nd, M9/39L, are the same as in (**A**). MT: wild-type metacyclic trypomastigotes from clone Dm28c (1 × 10^5 equivalent). Protein molecular weight standards (kDa) are indicated on the left.

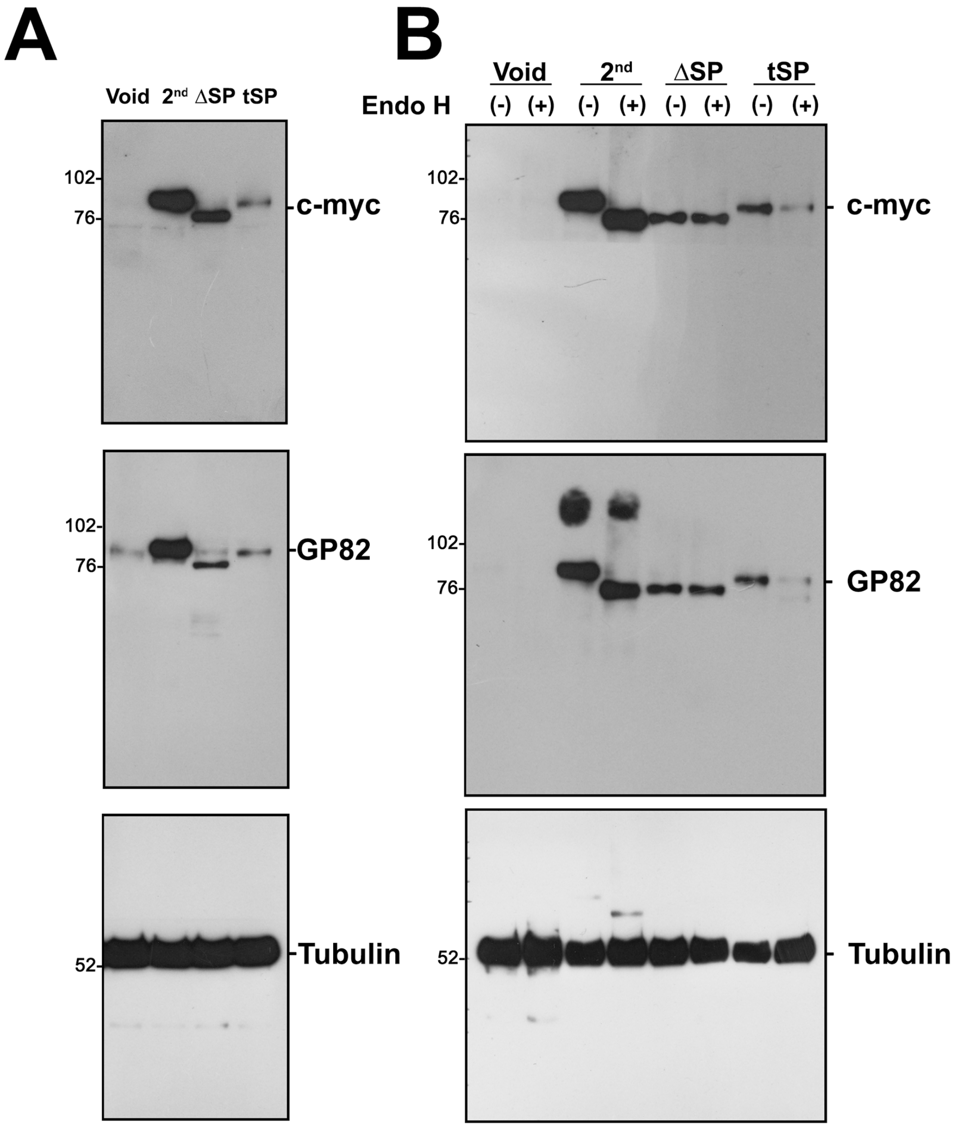

Figure S6. Full-length immunoblots from figure 3. A) Total protein extracts from transfected epimastigotes (6×10^6) were resolved on 10% SDS-PAGE, transferred to nitrocellulose membranes and incubated with 9E10 (anti-c-myc), 3F6 (anti-GP82) or anti-tubulin monoclonal antibodies as described in Fig. S6. **B)** Total protein extracts from transfected epimastigotes (6×10^6) were treated (+) or mock treated (-) with 500 U of endoglycosidase H (Endo H) at 37 °C for 3 h. Samples were separated on 10% SDS-PAGE, transferred to nitrocellulose membranes and incubated with anti-c-myc (upper panel) or anti-GP82 (lower panel) monoclonal antibodies. Void: epimastigotes transfected with empty pTEX plasmid; 2nd: transfected parasites carrying the pTEX-2nd construct; ΔSP: epimastigotes transfected with pTEX-ΔSP construct (without SP); tSP: transfected parasites carrying the pTEX-tSP construct (transposed SP). Protein molecular weight standards (kDa) are indicated on the left.

Table S1. Oligonucleotides used in this study

	Oligonucleotide	Sequence (5’→ 3’)*
1	4G6stF	*TCTAGA*ATGCTCTCACGTGTTGCT
2	4G6ndF	*GGATCC*ATGTCCCGGCGTGTGTTT
3	82MatBF	*GGATCC*ATGAAGGAGAATGATGGCAAA
4	cmyNF	**AAAAGCTAATTTCT**GAGGAAGACCTATGGGTCAATCTAT
5	cmyNR	**AGAAATTAGCTTTT**GCTCAGCACTTCTTAAATCACT
6	82p4R	CGTCTTCAGCTCCTCGGTCAGGCG
7	P82HinR	GGG*AAGCTT*TCAGTAAAGGGCCGCAAAGC
8	MtoLF	TCACGTGTTGCTGCAGTGTTGGCA
9	M2toLF	**AGGCCCAACTTGTCCCGG**
10	M2toLR	**CCGGGACAAGTTGGGCCT**
11	1stMtoLXbF	*TCTAGA*ATGCTCTCACGTGTTGCTGCAGTG
12	38WVNF	**AGGCCCAACTGGGTCAAT**CTATTTGTG
13	WNV38R	**ATTGACCCAGTTGGGCCT**CTGCCGCTC
14	dipSPF	**GGTGTTGTGATGTCCCGG**CGTGTGTTT
15	SPdipR	**CCGGGACATCACAACACC**TGAGCCACC
16	mycMEDF	**GAAGACCTAATGGAGGAT**GGCACCATT
17	MEDmycR	**ATCCTCCATTAGGTCTTC**CTCAGAAAT

* Restriction sites, start/stop codons and mutated codons are denoted in italic, underlined and double underlined, respectively. Bold nucleotides indicate complementary regions between oligonucleotides 4 and 5; 9 and 10; 12 and 13; 14 and 15, and 16 and 17.

Supplementary information S1. Deduced amino acid sequences of GP82 constructs generated in this study. Predicted signal peptide (red) and GPI-anchor addition signal (black) are highlighted in bold and italic bold fonts, respectively. The putative GPI-anchor acceptor amino acid (ω) is highlighted in yellow. C-myc epitope is indicated in underlined bold font (blue). Predicted *N*-glycosylation sequons are indicated in underlined italic font. Protein sequences of clones 5.4G6 (ABR19835) and J18 (AAA21303) are included for comparative purposes. Coloured amino acids follow the colour code of Fig. 1B in the main manuscript.

1st
MLSRVAAVMAPRTHNRRRVTGSSGRRREGGESERQRPN**MSRRVFDSTILLLLVTTMCCDTCGAAA**AKEND
GKSDLRSA**EQKLISEEDL**WVNLFVPQTTPVLPEGGGTPGTKRDAFVSPSLVSAGGVLAAFARGEIDAQYA
VDGKLIKPTSSAVVAEYIDSSWDWFTLVEKVSESTWKAYTVLSKAEGKGNLDVVLSPTTTMKGNKVFLLV
GSYDML*NES*GIWKRDSPDLKLVVGEVTKPSAGGEPSGWITWGTPTSL*NQT*TLKIPKAGLKDFYSSGGSGV
VMEDGTIVFPVIAFNAGNAGFSTTIYSTDDGANWMLS*NGT*PPAECLEPRITEWEGSLPMIVDCVDGQRVY
ESRDMGTTWTEAVGTLSGVWAKSQSFFRDLNLRVDALIAATIEGRKVMLYTQRGYASGEKRVNPLYLWVT
DN*NRS*FYFGPIAMGNAANSMFVSSLLYSDGSLHLLQRRANDKGSVISLARLTEELKTIKSVLSTWSKLDA
SFSASSTPTAGLVGLLSNSASGDAWIDDYRSVNAKVMNAVKVHDGFKFTGFGSGAIWPVNNRESNGPHTF
VNY*NFT*LVATVIVHKVPK*NST*TLLGAVLAEPISTLFIGLSYGTDGTWETVFNGETTTSGSTWMPGKEYQV
ALMLQDGNKGSVYVDGMSVGSLATLPTPEVRGAEIADFYFVGGEDEEDKKSSSVTVKNVFLYNRPLGADE
LRMVKKI**D*****GSMHGGVSRALLLLLGLCGFAALY***

2nd
MSRRVFDSTILLLLVTTMCCDTCGAAAAKENDGKSDLRSA**EQKLISEEDL**WVNLFVPQTTPVLPEGGGTP
GTKRDAFVSPSLVSAGGVLAAFARGEIDAQYAVDGKLIKPTSSAVVAEYIDSSWDWFTLVEKVSESTWKA
YTVLSKAEGKGNLDVVLSPTTTMKGNKVFLLVGSYDML*NES*GIWKRDSPDLKLVVGEVTKPSAGGEPSGW
ITWGTPTSL*NQT*TLKIPKAGLKDFYSSGGSGVVMEDGTIVFPVIAFNAGNAGFSTTIYSTDDGANWMLS*N*
*GT*PPAECLEPRITEWEGSLPMIVDCVDGQRVYESRDMGTTWTEAVGTLSGVWAKSQSFFRDLNLRVDALI
AATIEGRKVMLYTQRGYASGEKRVNPLYLWVTDN*NRS*FYFGPIAMGNAANSMFVSSLLYSDGSLHLLQRR
ANDKGSVISLARLTEELKTIKSVLSTWSKLDASFSASSTPTAGLVGLLSNSASGDAWIDDYRSVNAKVMN
AVKVHDGFKFTGFGSGAIWPVNNRESNGPHTFVNY*NFT*LVATVIVHKVPK*NST*TLLGAVLAEPISTLFIG
LSYGTDGTWETVFNGETTTSGSTWMPGKEYQVALMLQDGNKGSVYVDGMSVGSLATLPTPEVRGAEIADF
YFVGGEDEEDKKSSSVTVKNVFLYNRPLGADELRMVKKI**D*****GSMHGGVSRALLLLLGLCGFAALY***

M9/39L (in pink: engineered M→L substitutions)
MLSRVAAVLAPRTHNRRRVTGSSGRRREGGESERQRPN**LSRRVFDSTILLLLVTTMCCDTCGAAA**AKEND
GKSDLRSA**EQKLISEEDL**WVNLFVPQTTPVLPEGGGTPGTKRDAFVSPSLVSAGGVLAAFARGEIDAQYA
VDGKLIKPTSSAVVAEYIDSSWDWFTLVEKVSESTWKAYTVLSKAEGKGNLDVVLSPTTTMKGNKVFLLV
GSYDML*NES*GIWKRDSPDLKLVVGEVTKPSAGGEPSGWITWGTPTSL*NQT*TLKIPKAGLKDFYSSGGSGV
VMEDGTIVFPVIAFNAGNAGFSTTIYSTDDGANWMLS*NGT*PPAECLEPRITEWEGSLPMIVDCVDGQRVY
ESRDMGTTWTEAVGTLSGVWAKSQSFFRDLNLRVDALIAATIEGRKVMLYTQRGYASGEKRVNPLYLWVT
DN*NRS*FYFGPIAMGNAANSMFVSSLLYSDGSLHLLQRRANDKGSVISLARLTEELKTIKSVLSTWSKLDA
SFSASSTPTAGLVGLLSNSASGDAWIDDYRSVNAKVMNAVKVHDGFKFTGFGSGAIWPVNNRESNGPHTF
VNY*NFT*LVATVIVHKVPK*NST*TLLGAVLAEPISTLFIGLSYGTDGTWETVFNGETTTSGSTWMPGKEYQV
ALMLQDGNKGSVYVDGMSVGSLATLPTPEVRGAEIADFYFVGGEDEEDKKSSSVTVKNVFLYNRPLGADE
LRMVKKI**D*****GSMHGGVSRALLLLLGLCGFAALY***

ΔSP
MAKENDGKSDLRSA**EQKLISEEDL**WVNLFVPQTTPVLPEGGGTPGTKRDAFVSPSLVSAGGVLAAFARGE
IDAQYAVDGKLIKPTSSAVVAEYIDSSWDWFTLVEKVSESTWKAYTVLSKAEGKGNLDVVLSPTTTMKGN
KVFLLVGSYDML*NES*GIWKRDSPDLKLVVGEVTKPSAGGEPSGWITWGTPTSL*NQT*TLKIPKAGLKDFYS
SGGSGVVMEDGTIVFPVIAFNAGNAGFSTTIYSTDDGANWMLS*NGT*PPAECLEPRITEWEGSLPMIVDCV
DGQRVYESRDMGTTWTEAVGTLSGVWAKSQSFFRDLNLRVDALIAATIEGRKVMLYTQRGYASGEKRVNP
LYLWVTDN*NRS*FYFGPIAMGNAANSMFVSSLLYSDGSLHLLQRRANDKGSVISLARLTEELKTIKSVLST
WSKLDASFSASSTPTAGLVGLLSNSASGDAWIDDYRSVNAKVMNAVKVHDGFKFTGFGSGAIWPVNNRES
NGPHTFVNY*NFT*LVATVIVHKVPK*NST*TLLGAVLAEPISTLFIGLSYGTDGTWETVFNGETTTSGSTWMP
GKEYQVALMLQDGNKGSVYVDGMSVGSLATLPTPEVRGAEIADFYFVGGEDEEDKKSSSVTVKNVFLYNR
PLGADELRMVKKI**DGSMHGGVSRALLLLLGLCGFAALY**

tSP (in light blue: unexpected V→A substitution)
MLSRVAAVMAPRTHNRRRVTGSSGRRREGGESERQRPNWVNLFVPQTTPVLPEGGGTPGTKRDAFVSPSL
VSAGGVLAAFARGEIDAQYAVDGKLIKPTSSAVVAEYIDSSWDWFTLVEKVSESTWKAYTVLSKAEGKGN
LDVVLSPTTTMKGNKVFLLVGSYDML*NES*GIWKRDSPDLKLVVGEVTKPSAGGEPSGWITWGTPTSL*NQT*
TLKIPKAGLKDFYSSGGSGVa**MSRRVFDSTILLLLVTTMCCDTCGAAA**AKENDGKSDLRSA**EQKLISEED**
LMEDGTIVFPVIAFNAGNAGFSTTIYSTDDGANWMLS*NGT*PPAECLEPRITEWEGSLPMIVDCVDGQRVY
ESRDMGTTWTEAVGTLSGVWAKSQSFFRDLNLRVDALIAATIEGRKVMLYTQRGYASGEKRVNPLYLWVT
DN*NRS*FYFGPIAMGNAANSMFVSSLLYSDGSLHLLQRRANDKGSVISLARLTEELKTIKSVLSTWSKLDA
SFSASSTPTAGLVGLLSNSASGDAWIDDYRSVNAKVMNAVKVHDGFKFTGFGSGAIWPVNNRESNGPHTF
VNY*NFT*LVATVIVHKVPK*NST*TLLGAVLAEPISTLFIGLSYGTDGTWETVFNGETTTSGSTWMPGKEYQV
ALMLQDGNKGSVYVDGMSVGSLATLPTPEVRGAEIADFYFVGGEDEEDKKSSSVTVKNVFLYNRPLGADE
LRMVKKI**DGSMHGGVSRALLLLLGLCGFAALY**

ABR19835 (clone 5.4G6)
MLSRVAAVMAPRTHNRRRVTGSSGRRREGGESERQRPN**MSRRVFDSTILLLLVTTMCCDTCGAAA**AKEND
GKSDLRSAEELQWVNLFVPQTTPVLPEGGGTPGTKRDAFVSPSLVSAGGVLAAFARGEIDAQYAVDGKLI
KPTSSAVVAEYIDSSWDWFTLVEKVSESTWKAYTVLSKAEGKGNLDVVLSPTTTMKGNKVFLLVGSYDML
*NES*GIWKRDSPDLKLVVGEVTKPSAGGEPSGWITWGTPTSL*NQT*TLKIPKAGLKDFYSSGGSGVVMEDGT
IVFPVIAFNAGNAGFSTTIYSTDDGANWMLS*NGT*PPAECLEPRITEWEGSLPMIVDCVDGQRVYESRDMG
TTWTEAVGTLSGVWAKSQSFFRDLNLRVDALIAATIEGRKVMLYTQRGYASGEKRVNPLYLWVTDN*NRS*F
YFGPIAMGNAANSMFVSSLLYSDGSLHLLQRRANDKGSVISLARLTEELKTIKSVLSTWSKLDASFSASS
TPTAGLVGLLSNSASGDAWIDDYRSVNAKVMNAVKVHDGFKFTGFGSGAIWPVNNRESNGPHTFVNY*NFT*
LVATVIVHKVPK*NST*TLLGAVLAEPISTLFIGLSYGTDGTWETVFNGETTTSGSTWMPGKEYQVALMLQD
GNKGSVYVDGMSVGSLATLPTPEVRGAEIADFYFVGGEDEEDKKSSSVTVKNVFLYNRPLGADELRMVKK
I**DGSMHGGVSRALLLLLGLCGFAALY**

AAA21303 (clone J18)
ML*NES*GIWKRDSPDLKLVVGEVTKPSAGGEPSGWITWGTPTSL*NQT*TLKIPKAGLKDFYSSGGSGVVMED
GTIVFPVIAFNAGNAGFSTTIYSTDDGANWMLS*NGT*PPAECLEPRITEWEGSLPMIVDCVDGQRVYESRD
MGTTWTEAVGTLSGVWAKSQSFFRDLNLRVDALIAATIEGRKVMLYTQRGYASGEKRVNPLYLWVTDN*NR*
*S*FYFGPIPMGNAANSMFVSSLLYSDGSLHLLQRRANDKGSVISLARLTEELKTIKSVLSTWSKLDASFSA
SSTPTAGLVGLLSNSASGDAWIDDYRSVNAKVMNAVKVHDGFKFTGFGSGAIWPVNNRESNGPHTFVITF
TLCDVIVHKVPK*NST*TLLGAVLAEPISTLFIGLSYGTDGTWETVFNGETTTSGSTWMPGKEYQVALMLQD
GNKGSVYVDGMSVGSLATLPTPEVRGAEIADFYFVGGEDEEDKKSSSVTVKNVFLYNRPLGADELRMVKK
I**DGSMHGGVSRALLLLLGLCGFAALY**

References

1. Clemente, T. M., Cortez, C., Novaes Ada, S. & Yoshida, N. Surface Molecules Released by *Trypanosoma cruzi* Metacyclic Forms Downregulate Host Cell Invasion. *PLoS Negl. Trop. Dis*. **10,** e0004883; 10.1371/journal.pntd.0004883 (2016)
2. Cortez, M. *et al*. Infection by *Trypanosoma cruz*i metacyclic forms deficient in gp82 but expressing a related surface molecule, gp30. *Infect Immun*. **71**, 6184-6191 (2003)
3. Cortez, C. *et al*. Differential infectivity by the oral route of *Trypanosoma cruzi* lineages derived from Y strain. *PLoS Negl. Trop. Dis*. **6,** e1804; 10.1371/journal.pntd.0001804 (2012)
4. Cordero, E. M. *et al*. Proteomic analysis of detergent-solubilized membrane proteins from insect-developmental forms of *Trypanosoma cruzi*. *J. Proteome Res*. **8,** 3642-3652 (2009)
